# Jianpi Huayu Decoction Attenuates the Immunosuppressive Status of H_22_ Hepatocellular Carcinoma-Bearing Mice: By Targeting Myeloid-Derived Suppressor Cells

**DOI:** 10.3389/fphar.2020.00016

**Published:** 2020-02-18

**Authors:** Yingjie Xie, Yuan Zhang, Xiaohan Wei, Cheng Zhou, Yajing Huang, Xingwang Zhu, Yongxu Chen, Huihong Wen, Xuhui Huang, Juze Lin, Ziying Wang, Yan Ren, Baochao Fan, Xue Deng, Wei Tan, Changjun Wang

**Affiliations:** ^1^ School of Traditional Chinese Medicine, Southern Medical University, Guangzhou, China; ^2^ Guangdong Provincial People's Hospital, Guangdong Academy of Medical Sciences, Guangdong Geriatric Institute, Guangzhou, China; ^3^ School of Medicine, South China University of Technology, Guangzhou, China; ^4^ Guangzhou University of Chinese Medicine, Guangzhou, China

**Keywords:** Jianpi Huayu decoction, hepatocellular carcinoma, immunosuppression, myeloid-derived suppressor cells, maturation, immunotherapy, traditional Chinese medicines

## Abstract

Tumor-induced immunosuppressive microenvironment in which myeloid-derived suppressor cells (MDSCs) plays an important role, remains an obstacle for effective oncotherapy currently. Inducing MDSCs into maturation was confirmed as an effective method to reduce the tumor-bearing host's immunosuppression. Traditional Chinese medicines (TCM) possess characteristics of alleviating immunosuppression of cancer patients and low toxicity. Jianpi Huayu Decoction (JHD) was an experienced formula of TCM for oncotherapy based on TCM theory and clinical practice. We previously observed that JHD attenuated the expression of interleukin-10 (IL-10) and transforming growth factor beta (TGF-β) in tumor. IL-10 and TGF-β were found to be cytokines positively related to immunosuppression induced by MDSCs. Here, our study was designed to further investigate the regulation of JHD on the immune system in the H_22_ liver-cancer mouse model. Mainly, flow cytometry was used to detect the proportion of immune cells, to analyze the apoptosis, differentiation and reactive oxygen species of MDSCs. We found that JHD significantly reduced the destruction of spleen structure, reduced the proportion of regulatory T cells (Treg) and T helper 17 cells (Th17), and increased the proportion of cytotoxic T lymphotes (CTL), Dendritic cells (DC) and CD11b^+^Gr-1^+^cells in spleen, but with no significant change of T helper 1 cells (Th1), T helper 2 cells (Th2) and macrophages. In vitro experiments showed that apoptosis of MDSCs was decreased as the time of JHD stimulation increased, which partly explained the increase of CD11b^+^Gr-1^+^cells in the spleen. Meanwhile, JHD could promote the differentiation of MDSCs into macrophages and dendritic cells, attenuate expression of ROS in MDSCs and reduce its inhibition on the proliferation of CD4^+^ T cells, in vitro. Therefore, that the proportion of CD11b^+^Gr-1^+^ cells increased in the spleen of tumor-bearing hosts may not be villainy after treatment, when these drugs suppress the immunosuppressive ability of CD11b^+^Gr-1^+^ cells and promote it mature to replenish dendritic cell, at the same time. Generally, JHD may be a complementary and alternative drug for attenuating the immunosuppressive status induced by hepatocellular carcinoma, possibly by promoting differentiation and inhibiting the immunosuppressive activity of MDSCs.

## Introduction

Tumor-elicited immunosuppression remains a key obstacle for the immunotherapy of hepatocellular carcinoma (HCC) (Xie et al., 2018). Myeloid-derived suppressor cells (MDSCs) is a group of immature immunosuppressive cells, and could be divided into monocytic and granulocytic subsets according to surface marker and morphological characteristic (Safari et al., 2019). But its process to differentiate into antigen-presenting cells (APC) was inhibited under the conditions of tumorigenesis and chronic inflammation. MDSCs played many important roles, e.g. promoting tumor angiogenesis, mediating resistance of chemotherapeutic agents, and especially participating in immunosuppression (Park and Youn, 2019).

Finding out effective ways to inhibit tumorigenic effect of MDSCs was of great significance in ameliorating the progression and relapse of tumor (Rivoltini et al., 2019). Inhibiting activation, inducing differentiation into maturation, promoting apoptosis and reducing infiltration into tumor were found to be the main methods (Anani and Shurin, 2017). However, drugs for inducing MDSCs to mature received more researches, e.g. oxaliplatin (Kim and Kim, 2019), all-trans retinoic acid (Tobin et al., 2018), resveratrol (Zhao et al., 2018a).

Pharmacological and clinical researches had confirmed that traditional Chinese medicines (TCM) were capable of inhibiting progression of tumor, ameliorating immunosuppression and chemotherapeutic agents resistance, and improving the quality of life and prolonging survival time of cancer patient (Yan et al., 2017). TCM intervention could be an appropriate choice for advanced liver-cancer patients who could not undergo radical surgery. Jianpi Huayu Decoction (JHD), which was clinically used for the alternative and complementary therapy of liver-cancer, consists of Citrus medica L., Curcuma phaeocaulis Valeton, Atractylodes macrocephala Koidz., Sophora flavescens Aiton, Wolfiporia cocos (Schwein.) Ryvarden et Gilb., and Hedyotis diffusa Willd. JHD had been confirmed with anti-hepatoma effects that reduced the viability of liver-cancer cells and a high dose of JHD provided the best curative effects (Lin et al., 2017). We also observed that JHD attenuated the expression of interleukin-10 (IL-10) and transforming growth factor-beta (TGF-β) in hepatic tumour. IL-10 and TGF-β were found to be cytokines involved in MDSCs-induced immunosuppression. However, the regulatory effect of JHD on immunosuppressive cells such as MDSCs remained unclear. Therefore, this study was designed to further investigate the effects of JHD on the immune system in a liver-cancer mouse model.

## Materials and Methods

### Cells and Culture

The H_22_ hepatoma carcinoma cell line was kindly provided by Stem Cell Bank, Chinese Academy of Sciences (Shanghai, China) and were cultured in RPMI-1640 medium supplemented with 10% fetal bovine serum (FBS) at 37°C in a 5% CO_2_ atmosphere. MDSCs, which were isolated from the bone marrow and spleens of H_22_ hepatoma carcinoma-bearing mice, were cultured in RPMI-1640 medium supplemented with 10% FBS and 20 ng/mL recombinant mouse granulocyte-macrophage colony-stimulating factor (GM-CSF, PeproTech). The purity of the MDSCs was up to 94% (**Supplementary Material 1**).

### Preparation of Jianpi Huayu Decoctionextracts

Dried *Citrus medica L.* (*Citrus medica Linn. var. sarcodactylis* (*Noot.*) *Swingle*, *Rutaceae*, batch number: 180902031), *Curcuma phaeocaulis Valeton* (*Curcuma zedoaria* (*Christm*.) *Rosc*, *Zingiberaceae*, batch number: 181202941),* Atractylodes macrocephala Koidz*. (*Atractylodes macrocephala Koidz*, *Compositae*, batch number: 181003941), *Sophora flavescens Aiton* (*Sophora flavescens Alt*, *Leguminosae*, batch number: 181003011), *Wolfiporia cocos* (*Schwein*.) *Ryvarden et Gilb*. (*Poria cocos* (*Schw.*) *Wolf*, *Polyporaceae*, batch number: 18110671) (Wei et al., 2016; Gao et al., 2019), and *Hedyotis diffusa Willd*. (*Hedyotis diffusa Willd. Var. Longipes Nakai*, *Rubiaceae*, batch number: 181103361) purchased from Guangdong Provincial People's Hospital (supplied by KANGMEI PHARMACEUTICAL CO., LTD (Guangdong, China, Drug GMP certificate: GD20180882, Drug Manufacturing Certificate: GD20160335)) were added together at a ratio of 3:3:3:5:5:5, respectively, to generate solutions of JHD extracts. Above herbs had been authenticated and confirmed to the quality standards of Chinese pharmacopoeia (2015 version), which included the morphological identification, microscopic identification, chemical identification and thin-layer chromatography (**Supplementary Material 2**). To prepare water extracts of JHD, the herb mixture was blended into double-distilled water (1:10, Weight/Volume) for 30 min and then heated for 1 hour. Then water extracts were evaporated to 4g/ml herb water extract. Partial water extract was processed into lyophilized powder. All the extracts were stored at -80°C until used.

### Animal Model and Treatment

Twenty (20) male BALB/c mice (six to eight weeks-old, 18~22g) were purchased from Animal Center of Southern Medical University (Guangzhou, China), and maintained under specific pathogen-free (SPF) conditions, at 25 ± 1°C, relative humidity of 55 ± 5%, and a 12h/12h light/dark cycle. To establish subcutaneous tumor-bearing mice, 8 × 10^^5^ H_22_ hepatoma carcinoma cells suspended in 50μl of phosphate buffer solution (PBS) were injected into the right flank of ten mice, and the mice were randomly divided into JHD-treated (n=5) and PBS-treated (n = 5) groups. Mice in the JHD-treated group were orally administered JHD extract [24.96g/kg (Lin et al., 2017)] per day continuously for 12 days after the second day of injection, and mice in the PBS-treated group were treated with an equal volume of PBS as a control. The length and width of the subcutaneous tumors were measured, and the mice were weighed daily. Besides, ten male BALB/c mice were used to establish an in situ hepatocellular carcinoma-bearing model. Briefly, the mice were anaesthetized and operated in aseptic conditions. Approximately 1 × 10^^6^ H_22_ hepatocellular carcinoma cells suspended in 5μl of PBS were injected into the edge of the right lobe of the liver. After suturing the abdominal muscles and skin, the mice were fed in SPF conditions for 15 days. All animal procedures were approved by the Institutional Animal Care and Use Committee of Southern Medical University (Guangzhou, China).

### HPLC Analysis of JHD

**Figure 1:**
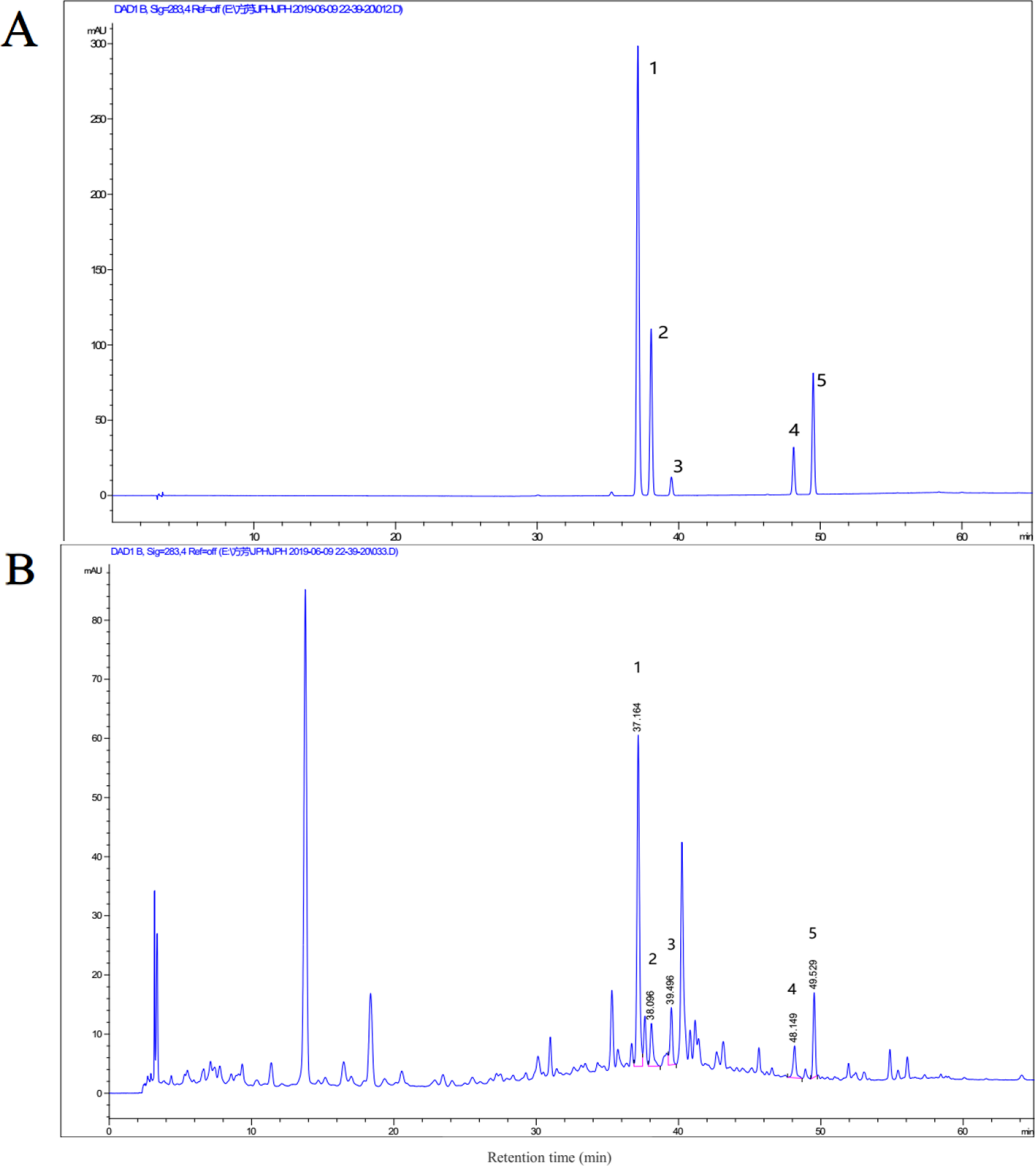
High-performance liquid chromatography (HPLC) analysis of Jianpi Huayu Decoction (JHD). HPLC chromatogram of mixed reference substance was shown at **(A)** and that of JHD extracts was shown at **(B)**. The term and concentration of five monomers in JHD extraction were identified and shown: 1. p-coumaric acid (602.3419μg/g); 2. ferulic acid (187.500μg/g); 3. hesperidin (341.3894μg/g); 4. 5,7-dimethoxycoumarin (433.4916μg/g); 5. bergapten (555.2405μg/g).

To evaluate the quality and stability of the extracts of JHD, high-performance liquid chromatography (HPLC) analysis was performed by Agilent 1260 HPLC system (Agilent Technologies). The mobile phase system was composed of water with 0.05% phosphoric acid (A) and methanol (B). The gradient elution profile was: 0∼20min, 5%∼21% B; 20∼55 min, 21%∼88% B; 55∼65 min; 88% B. The extracts of JHD were separated by Agilent ZORBAX SB-C18 column (4.6 × 250 mm, 5 µm) at 30°C, with 1.0 ml/min of mobile phase flow rate. The UV spectrum was set at 283nm. Hesperidin (HPLC, > 98% purity, CAS:520-26-3), p-coumaric acid (HPLC, > 98% purity, CAS: 501-98-4), ferulic acid (HPLC, > 98% purity, CAS: 1135-24-6), 5,7-dimethoxycoumarin (HPLC, > 98% purity, CAS: 487-06-9) and bergapten (HPLC, > 98% purity, CAS: 484-20-8) were purchased from Chengdu Chroma-Biotechnology Co., Ltd (Chengdu, China). The reference substances were detected under the same condition of JHD. HPLC chromatograms of JHD is shown at **Figure 1**. Besides, the methodology analysis of JHD, which was included linear relation, stability, repeatability, precision and recovery test, was also performed, and the results were shown at **Supplementary Material 3**. Concentration of the identified monomer was the weight in the lyophilized powder per gram.

### Analyzing the Effect of JHD on MDSCs Viability Using Cell Counting kit-8

MDSCs from the spleen of in situ hepatocellular carcinoma-bearing mice were purified by magnetic-activated cell sorting (MACS, Miltenyi Biotec). The cells were counted, and 2 × 10^^3^ MDSCs in 99μl of culture medium were seeded into 96-well plates. And then 1μl of JHD extracts (0μg/ml, 62.5μg/ml, 125μg/ml, 250μg/ml, or 500μg/ml) were added. Each concentration was tested in three wells, and the control group was treated with 1μl of plain medium. After 24 hours of culture, 10μl of cell counting kit-8 (CCK-8) reagent (Glpbio) was added, and the cells were incubated for 30 min. A Multiscan Spectrum (Thermo Scientific) at a 450nm wavelength was used to detect absorbance values (OD values) (Zhao et al., 2019). Cell survival rate was equal to the value of [(OD of the JHD group - OD of the blank group)/(OD of the control group - OD of the blank group)] × 100%. The effect of viability of JHD on H_22_ cells was detected following the method above.

### Flow Cytometry (FCM)

To prepare single-cell suspensions from mouse spleens, half of each spleen was placed on a 70-μm cell filter, and rinsed with PBS while gently grinding the tissues with the inner core of a 1ml syringe. To obtain single-cell suspensions from mouse bone marrow, we used a 1ml syringe to flush the bone marrow cells from the femur and filtered them through a 70-μm cell filter. After centrifugation, the erythrocytes were lysed with Red Blood Cell Lysis Buffer (Solarbio) at 4°C for 5 minutes and washed once with 5% bovine serum albumin (BSA). Fc-receptor blocking reagent (Miltenyi Biotec) was used to block the Fc-receptors on cells, and a Percp/Cy5.5-CD45.2 antibody (Biolegend) marked nucleated hematopoietic cells, following the manufacturer's instructions (Yap et al., 2018). Allophycocyanin (APC)-CD11b, fluorescein isothiocyanate (FITC)-Gr-1, phycoerythrin (PE)-ly6G, FITC-Ly6C antibody (eBioscience) were used to identify MDSCs and their subgroups. FITC-CD11c and PE-F4/80 antibodies (Biolegend) were used to detect dendritic cells and macrophages, respectively. Cytotoxic T lymphotes (CTL) were detected with APC-CD8a and PE-interferon-γ (IFN-γ), Th1 were detected with APC-CD4 and PE-IFN-γ, Th2 were detected with APC-CD4 and PE-interleukin-4 (IL-4), Th17 were detected with APC-CD4 and PE-interleukin-17a (IL-17a), and Treg were detected with APC-CD4 and PE-forkhead box p3 (Foxp3). All samples were detected with Flow cytometry (Beckman Coulter cytoflex) using a gate of CD45.2^+^ cells. The data were analyzed by CytExpert software (Beckman). Blank and single staining tubes were set up for all tests, and the fluorescent compensation matrix was generated using CytExpert software.

### Analyzing the Effect of JHD on MDSCs Apoptosis Using Annexin-V-FITC/PI

MDSCs, which were isolated from the spleens of in situ H_22_ hepatocellular carcinoma-bearing mice by MACS technique (Miltenyi Biotec), were cultured in 24 well culture-plate and treated with either JHD (250μg/ml, 500μg/ml) or oxaliplatin (1μg/ml, Solarbio). After 24 hours or 36 hours of incubation, the cells were collected for Annexin-V-FITC/PI (KeyGEN BioTECH) staining following the manufacturer's instructions and detected by Flow cytometry (Beckman Coulter Cytoflex). Meanwhile, cells without fluorescence staining were used in the blank tube, and cells stained with Annexin-V-FITC or PI were used in single staining tubes. The fluorescent compensation matrix was conducted by CytExpert software.

### Analyzing the Effect of JHD on MDSCs Differentiation With FCM

MDSCs from the spleens of orthotopic H_22_ liver cancer-bearing mice were isolated by MACS technique (Miltenyi Biotec). Approximately 1 × 10^^6^ MDSCs were cultured in 24-well culture-plate and stimulated by JHD (250μg/ml, 500μg/ml) and oxaliplatin(1μg/ml, Solarbio) (Kim and Kim, 2019), respectively. After stimulating for 36 or 48 hours, cells were collected and their Fc-receptors were blocked with Fc-receptor blocking reagent (Miltenyi Biotec). The cells were then stained with APC-CD11c, PE-F4/80, FITC-CD80, and Percp/Cy5.5-MHCII antibodies (Biolegend) following the manufacturer's instructions and detected with Flow cytometry (Beckman Coulter Cytoflex). Cells not bound to fluorescent antibodies and cells bound to one fluorescent antibody were used for the fluorescent compensation which was performed by CytExpert software.

### Detection of Reactive Oxygen Species in MDSCs *In Vitro*

To detect the expression of Reactive Oxygen Species (ROS) in MDSCs cells, bone marrow cells from healthy BALB/c mice were isolated and co-cultured with recombinant mouse GM-CSF (10ng/ml, PeproTech), recombinant mouse interlrukin-6 (10ng/ml, PeproTech) and recombinant mouse IL-4 (10ng/ml, PeproTech) for 4 days, and collected. The cells were then exposed to JHD (0μg/ml, 250μg/ml, 500μg/ml) for 36 hours, collected, and incubated with 2′-7′-dichlorodihydroﬂuorescein diacetate (DCFH-DA, Beyotime). Then, Fc-receptor blocking reagent (Miltenyi Biotec) was used to block the Fc-receptor of cells at 4°C for 10min. Finally, the cells were stained with APC-CD11b and PE-Gr-1 antibodies (Biolegend) with reference to reagent specification and detected with Flow cytometry (Beckman Coulter Cytoflex). Cells without fluorescence staining were used in the blank tube, and cells stained with one fluorescent antibody were used in single staining tubes. The blank tube and single staining tubes were used for fluorescent compensation.

### Analysis of CD4^+^ T Lymphocyte Proliferation *In Vitro*

Naïve CD4^+^T cells were obtained from the spleens of healthy BALB/c mice using a naïve CD4^+^T cells isolation kit (Miltenyi Biotec) following the manufacturer's instruction. The cells were then labelled with carboxyl fluorescein diacetate succinimide (CFSE) and cultured in 24-well plates that were coated with anti-CD3 (10μg/ml, BioGems) and anti-CD28 (2μg/ml, BioGems) antibodies for 12 hours. Meanwhile, MDSCs were isolated from the spleen of in situ H_22_ hepatocellular carcinoma-bearing mice using MACS technique (Miltenyi Biotec) and pretreated with JHD (500μg/ml) or isochoric PBS for 36 hours. Subsequently, CD4^+^ T cells and MDSCs were collected respectively, and co-cultured at ratios of 1:1and 1:2. For the positive group, naive CD4^+^ T cells were co-cultured with DCs (CD11b^+^CD11c^+^) which were obtained by fluorescence-activated cell sorting (FACS). Naïve CD4^+^T cells, which were activated by anti-CD3 and anti-CD28 antibodies, were used as the negative control. After 48 hours of culture, the cells were collected and Fc-receptor blocking reagent (Miltenyi Biotec) was used to block the Fc-receptor of cells at 4°C for 10min. Finally, cells were incubated with a Percp/Cy5.5-CD4 antibody (eBioscience) at 4°C for 10min. The percentage of CFSE was detected by Flow cytometry (Beckman Coulter Cytoflex), with gate of Percp/Cy5.5-CD4^+^ cells.

### Hematoxylin and Eosin Staining of Spleen

Freshly-obtained mouse spleens were fixed in 4% paraformaldehyde, embedded into paraffin, cut into 4μm-thick slices. The slices were baked at 60°C for 2 hours. The slices were then dewaxed by dipping them into xylene, anhydrous ethanol and alcohol orderly. Next, the slices were dipped into hematoxylin reagent (Sangon Biotech) for 5 min, rinsed with distilled water for 5 min, and dehydrated with anhydrous ethanol for 5 min. Finally, the slices were dipped into an eosin dye solution (Sangon Biotech) for 5 min, dehydrated with anhydrous ethanol for 5 min, and sealed with neutral gum. All slices were observed under an inverted microscope and images were collected.

### Immunohistochemistry of Tumours and Spleens

Fresh tumor and spleen tissues were fixed in 4% paraformaldehyde at room temperature. After paraffin-embedding, the tissues were cut into 5μm-thick sections for immunohistochemistry staining. First, sections were deparaffinized, processed with an AutoFluo Quencher (Solarbio) and blocked with 5% BSA. Next, the sections were incubated with anti-CD11C, anti-F4/80, anti-inducible nitric oxide synthase (iNOS), and anti-STAT3 (signal transducer and activator of transcription-3) antibodies (Cell Signaling Technology) at 4°C overnight, and incubated with anti-IgG-Horseradish peroxidase (HRP) for 2 hours at room temperature. Finally, a DAB Immunohistochemistry Color Development kit (Sangon Biotech) was used for chromogenic reaction.

### Statistical Analysis

GraphPad Prism 5 was used for data statistics and mapping. Data is presented as the mean ± sem. Student's t-test was used to compare data between two groups. Statistically significant difference is assumed at *p-*value of <0.05.

## Results

### HPLC Chromatograms and Quality Control of JHD

HPLC analysis was conducted to evaluate the quality and stability of JHD. The methodology results, which included linear relation, stability, repeatability, precision and recovery test, showed that JHD had good repeatability and stability (**Supplementary Material 3**). HPLC chromatograms of mixed reference substance and was shown at **Figure 1A** and that of JHD extracts was shown at **Figure 1B**. Here, we identified five known monomers and its concentration in JHD lyophilized powder, including hesperidin (341.3894μg/g), p-coumaric acid (602.3419μg/g), ferulic acid (187.500μg/g), 5,7-dimethoxycoumarin (433.4916μg/g) and bergapten (555.2405μg/g). However, we did not detect the presence of atractylenolide-I, atractylenolide-III, curcumenol, oxymatrine and ursolic acid in the water extract of JHD (data not show).

### JHD Inhibited the Growth of Subcutaneous H22 Hepatocellular Carcinoma

**Figure 2:**
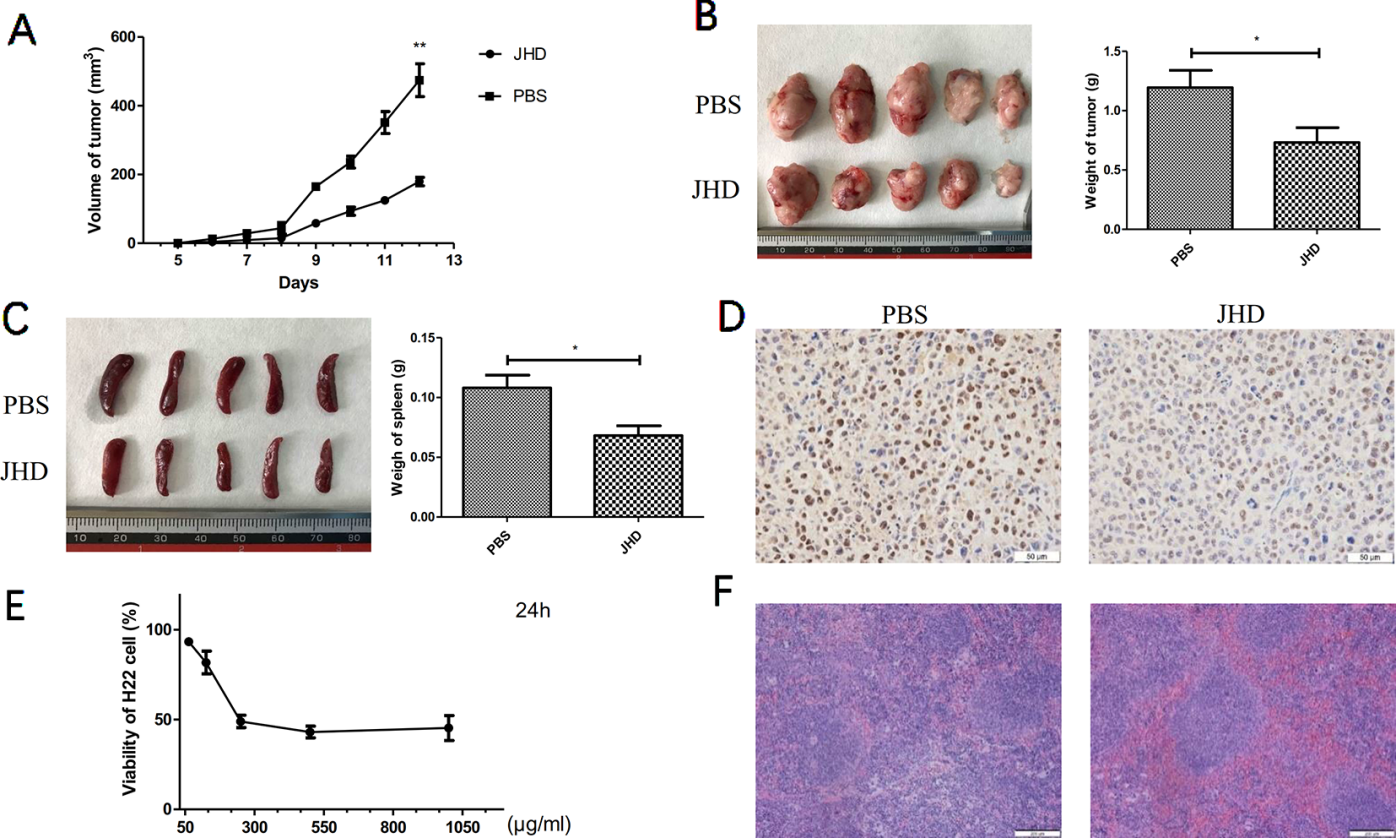
Jianpi Huayu Decoction (JHD) inhibited the growth of subcutaneous H_22_ hepatocellular carcinoma. 8 × 10^^5^ H_22_ hepatocellular carcinoma cells were injected subcutaneously into right flank of male BALB/c mice. Mice were randomly divided into PBS-group and JHD-group (n = 5). One day after injection, JHD (24.96 g/kg per day) were administered orally and the same volume of PBS was used as the control. **(A)** Volume of subcutaneous tumor were measured every day (n = 5). **(B)** Picture of subcutaneous tumor and its weight were shown (n = 5). **(C)** Spleen and its weight of mice were shown. **(D)** Representative pictures of PCNA immunohistochemical staining in tumor (×400 magnification, n = 5). **(E)** CCK-8 was used to detect the cell viability of H_22_ cells (n = 3). **(F)** Representative pictures of H&E staining of spleen (×400 magnification, n = 5). Scale bar = 50μm. *: *p*< 0.05; **: *p* < 0.01.

Our previous studies had demonstrated that a high-dose (24.96g/kg/day) of JHD could delay the progression of liver cancer, and reduce the expression levels of IL-10 and TGF-β in tumor (**Supplementary Data 4**). After 12 days of gavage, the growth of subcutaneous tumor was significantly delayed in JHD-treated group (**Figure 2A**), and tumor weight was lower than that of the PBS-control group (**Figure 2B**). The results of immunohistochemical staining of PCNA (**Figure 2D**) and CCK-8 cytotoxicity assay (**Figure 2E**) showed that the proliferation of H_22_ hepatocellular carcinoma cells was inhibited *in vivo* and in vitro. Besides, JHD could reduce the weight of spleen (**Figure 2C**) and the damage of spleen tissue structure (**Figure 2F**).

### JHD Increases the Proportion of CD11c^+^ and CD11b^+^Gr-1^+^Cells in Spleen

**Figure 3:**
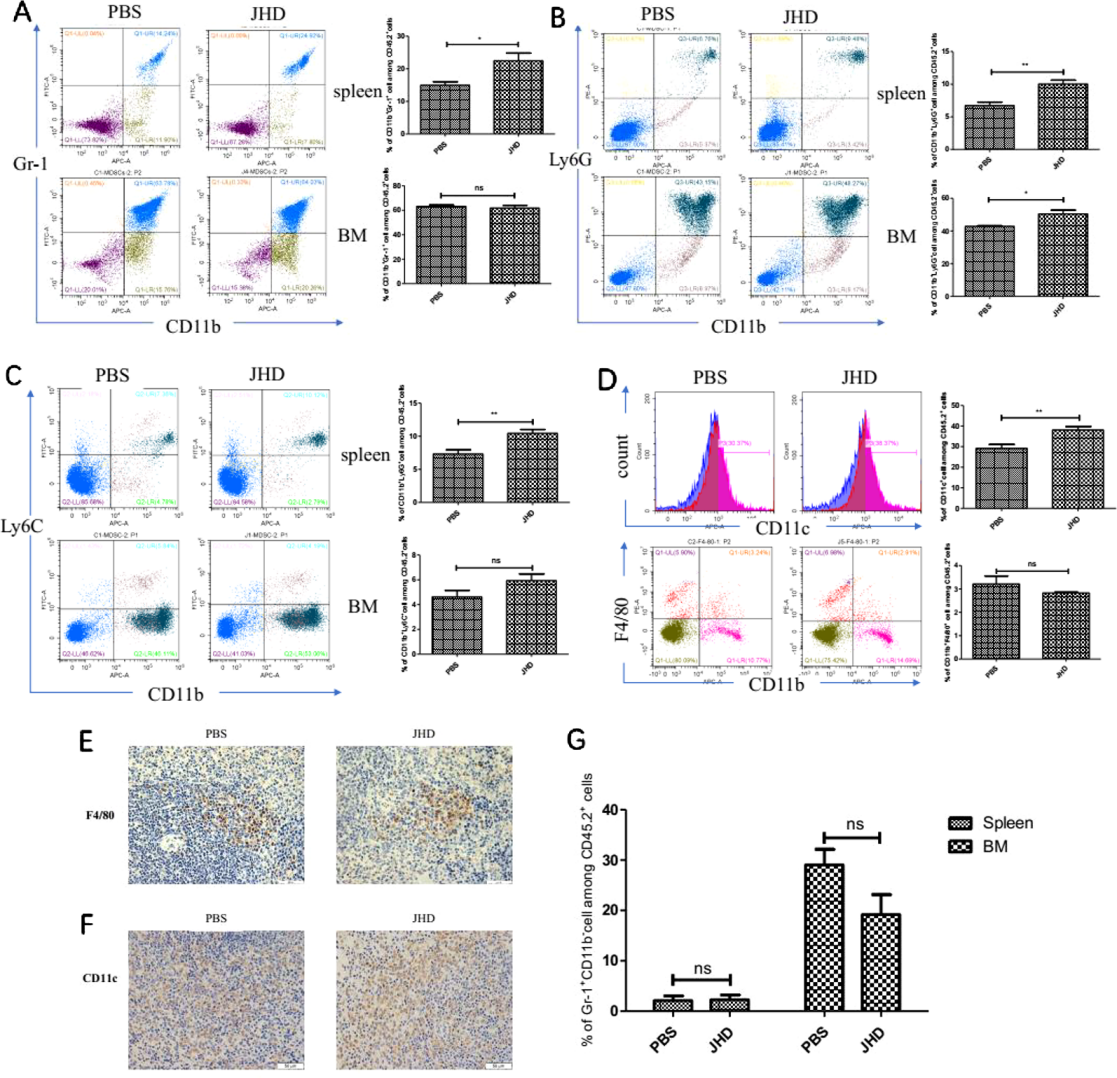
Jianpi Huayu Decoction (JHD) increases the proportion of CD11c^+^ and CD11b^+^Gr-1^+^cells in spleen. Subcutaneous tumor mouse models were established and administrated as described in **Figure 2**. Flow cytometry was performed on the percentage of Myeloid-derived suppressor cells (MDSCs) in spleen and bone marrow. Fc-R blocker was used to seal the cells before fluorescent antibody incubation, and CD45.2^+^ cells were gated. **(A)** The proportion of CD11b^+^ Gr-1^+^ cells in spleen and bone marrow were determined (n = 5). Representative flow cytometry data and statistical diagram are shown. **(B**, **C)** The proportion of CD11b^+^Ly6G^+^ cells and CD11b^+^Ly6C^+^ cells in spleen and bone marrow were determined. Representative flow cytometry data and statistical diagram are shown (n = 5). **(D)** The percentage of CD11c^+^ cells and CD11b^+^F4/80^+^ cells in spleen were analyzed, and shown by flow cytometry data and statistical diagram, respectively (n = 5). **(E**, **F)** Representative pictures of F4/80 and CD11c immunohistochemical staining in spleen were shown (×400 magnification, n = 5). **(G)** Statistical diagram of the percentage of Gr-1^+^ CD11b^-^ cells among CD45.2^+^ cells was shown (n = 5). *: *p*< 0.05; **: *p* < 0.01; ns, not significant.

Many antitumor drugs exhibited the abilities to reduce the accumulation of CD11b^+^Gr-1^+^ cells and immunosuppression of a tumor-bearing host (Kim and Kim, 2019). However, gemcitabine increased CD11b^+^Ly6C^high^ cells infiltration in bladder cancer tissues (Mu et al., 2019) and lenvatinib was associated with increased tumor-infiltrating and circulating CD11b^+^Gr-1^+^ cells (Gunda et al., 2019). In our research, we observed changes in the proportion of CD11b^+^Gr-1^+^ cells and subsets in the spleen and bone marrow, which were most relevant to recruitment and generation of MDSCs. In spleen, the proportion of both CD11b^+^Gr-1^+^ cells and its two subsets up-regulated after JHD treatment (**Figures 3A–C**). The proportion of CD11b^+^Gr-1^+^ cells and CD11b^+^Ly6G^-^Ly6C^+^cells showed insignificantly difference in bone marrow, but CD11b^+^Ly6G^-^Ly6^+^cells up-regulated after treated by JHD (**Figures 3A–C**). MDSCs were precursor cells of macrophage, dendritic cell and granulocyte. Here, we observed increased proportion of CD11c^+^ cells (**Figure 3D**), and insignificantly different proportion of CD11b^+^F4/80^+^ and Gr-1^+^CD11b^-^ cells (**Figures 3D**, **G**) in spleen of JHD-treated mice, which were also confirmed by immunohistochemistry (**Figures 3E** and **F**).

### JHD Reverses Imbalance of T Lymphocytes in Spleen of HCC-Bearing Mice

**Figure 4:**
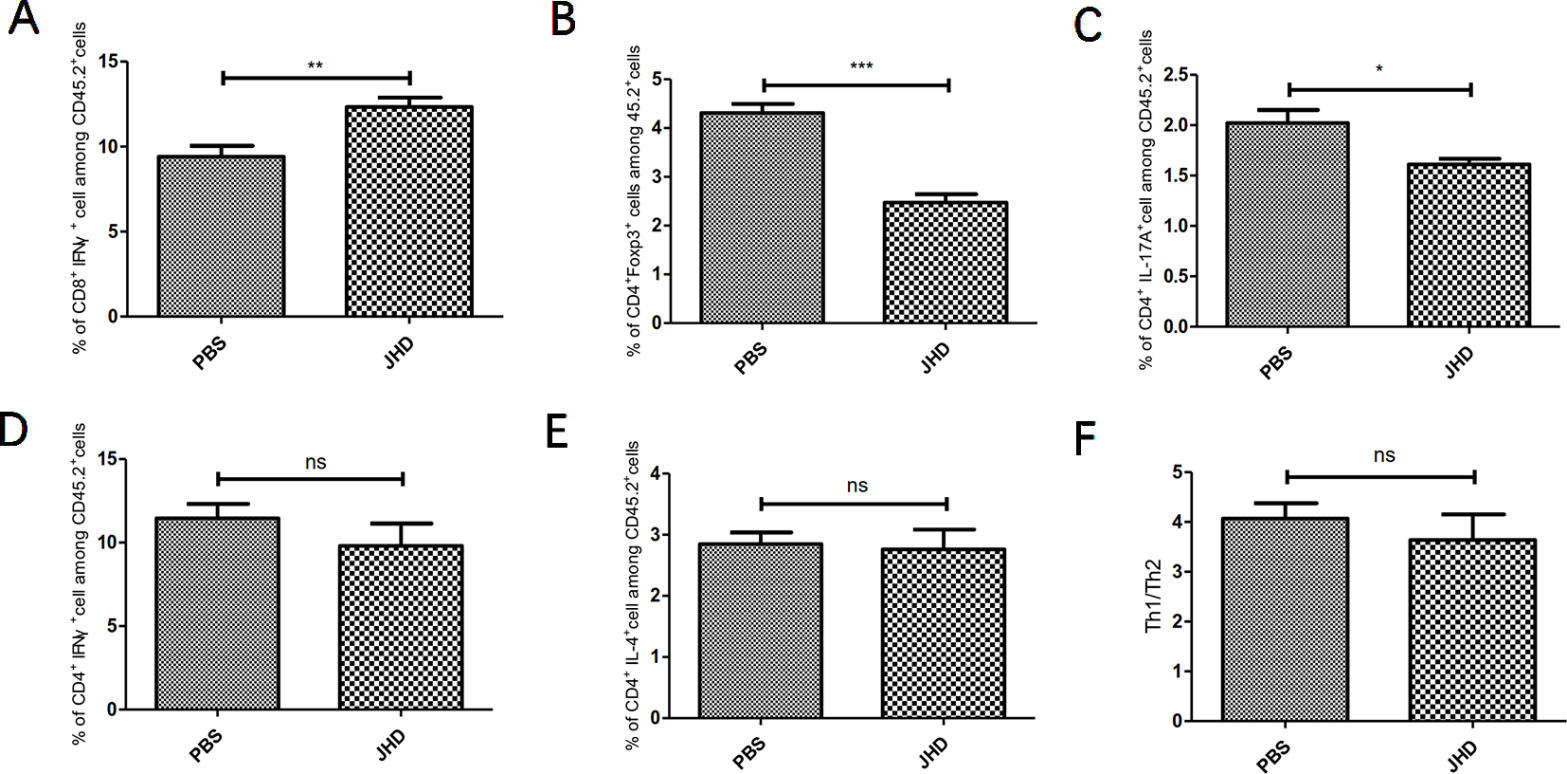
Jianpi Huayu Decoction (JHD) reverses imbalance of T lymphocytes in spleen of hepatocellular carcinoma bearing mice. Subcutaneous tumor mouse models were established as described in **Figure 2**. The mice were euthanized after 12 days of JHD continuous administration. Then flow cytometry was used to detect the cell subgroups in spleen. Fc-R blockers were used to seal the cells before fluorescent antibody incubation, and CD45.2^+^ cells were gated. **(A–E)** The percentage of CTL, Treg, Th17, Th1 and Th2 among CD45.2^+^ cells in spleen, respectively (n = 5). **(F)** The ratio of Th1/Th2 in spleen are shown (n = 5). *: *p*< 0.05; **: *p* < 0.01; ***: *p* < 0.001; ns, not significant.

The imbalance of T lymphocytes subsets played a crucial role in tumor development. As tumor progressed, MDSCs induced the expansion of T helper type 17 cell (Th17), T helper type 2 cell (Th2), regulatory T cell (Treg) and inhibited the proliferation and activation of cytotoxic T lymphocyte (CTL) and T helper type 1 cell (Th1), by secreting cytokines and immunosuppressive molecules (Sun et al., 2019). In this study, we found that JHD increased the percentage of CTL (**Figure 4A**) and decreased the percentage of Treg (**Figure 4B**) and Th17 (**Figure 4C**) cells, but did not affect that of Th1 (**Figure 4D**) and Th2 cells (**Figure 4E**) and Th1/Th2 ratio (**Figure 4F**). In general, JHD alleviated immunosuppression in H_22_ tumor-bearing mice.

### JHD Regulates Apoptosis of CD11b^+^Gr-1^+^ Cells *In Vitro*

**Figure 5:**
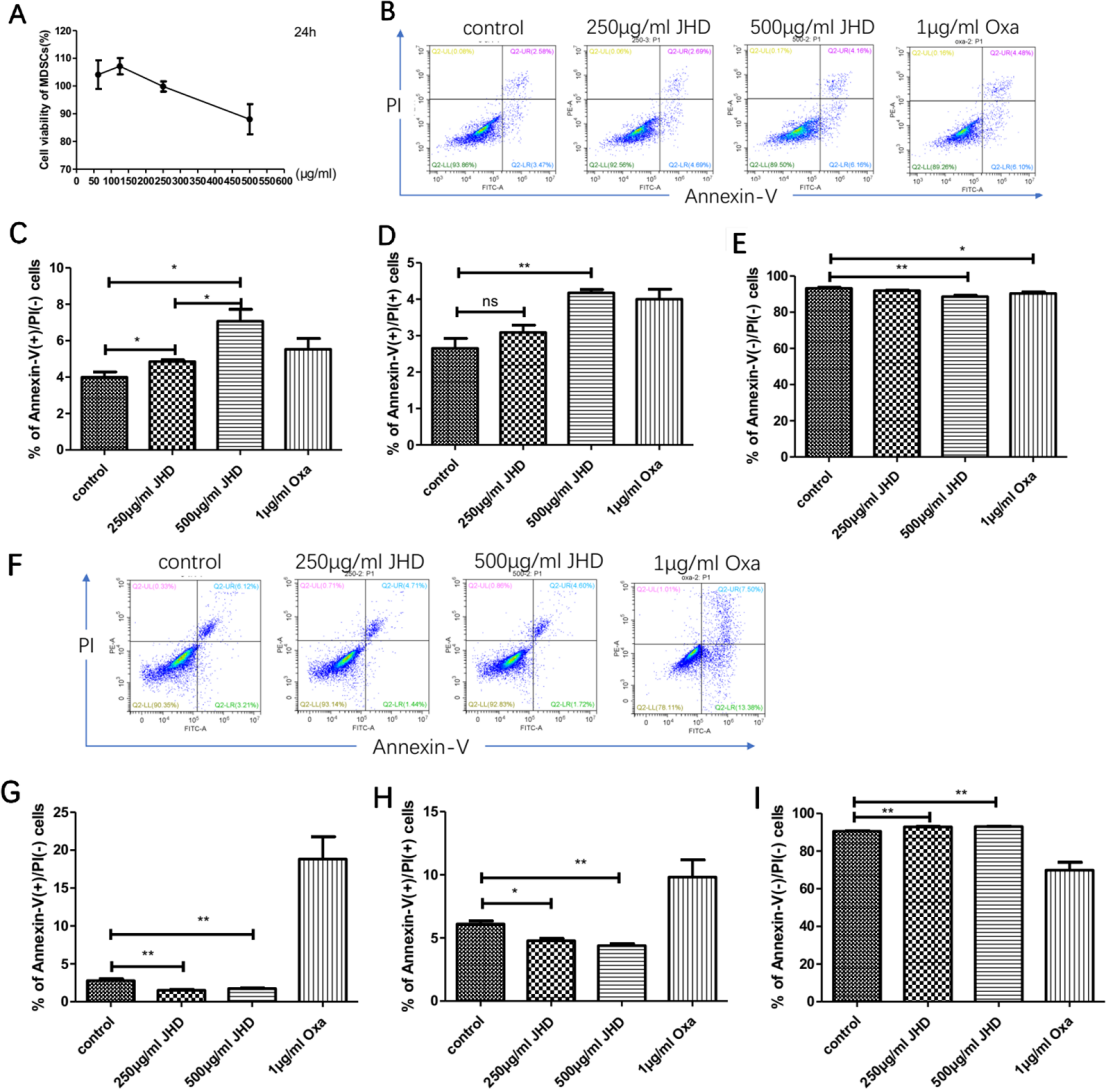
Jianpi Huayu Decoction (JHD) regulates apoptosis of CD11b^+^Gr-1^+^ in vitro. **(A)** The effect of JHD on cell viability of Myeloid-derived suppressor cells (MDSCs) which isolated from H_22_ hepatocellular carcinoma bearing mice was detected by Cell Counting Kit-8 (CCK-8) method (n = 3). Statistical diagram is shown. **(B–E)** MDSCs which isolated from H_22_ hepatocellular carcinoma bearing mice were stimulated by JHD (250μg/ml, 500μg/ml) or oxaliplatin (1μg/ml) in vitro, and the percentage of apoptosis cells was detected by flow cytometry at 24h (n = 3). Representative flow cytometry data and statistical diagrams are shown. And the percentage of non-apoptotic cell was shown at € (n = 3). **(F–I)** MDSCs were stimulated by JHD as above up to 36 hours, and the apoptosis cells was detected by flow cytometry (n = 3). And the percentage of non-apoptotic cell was shown at **(I)** (n = 3). *: *p*< 0.05; **: *p* < 0.01; ns, not significant.

The mechanism of increased splenetic CD11b^+^Gr-1^+^ cells after JHD treatment with JHD was not clear. CCL9 released by macrophages could recruit MDSCs from bone marrow to spleen (Li et al., 2019), but the percentage of CD11b^+^ F4/80^+^ macrophages in spleen was decreased after JHD treatment (**Figure 3D**). Therefore, we speculated that JHD might regulate the cell viability or apoptosis of MDSCs. In vitro, cell viability assay results demonstrated that when concentration was more than 250μg/ml, JHD began to inhibit MDSCs viability, and showed the opposite effect at less than 250μg/ml dose (**Figure 5A**). In addition, the IC50 value of JHD value was 653.9μg/ml. After 24h stimulation, JHD and oxaliplatin significantly induced early apoptosis of MDSCs (**Figure 5C**), meanwhile, the percentage of late apoptotic cells increased (**Figure 5D**) and that of living cells decreased (**Figure 5E**). Surprisingly, at 36 hours, 500μg/ml JHD significantly decreased apoptosis in cultured cells, compared with control group (**Figures 5F–I**). In general, the percentage of CD11b^+^Gr-1^+^ phenotypic cells in the JHD-treated group increased *in vivo*, possibly because of reduced apoptosis.

### JHD Promotes the Differentiation of MDSCs *In Vitro*

**Figure 6:**
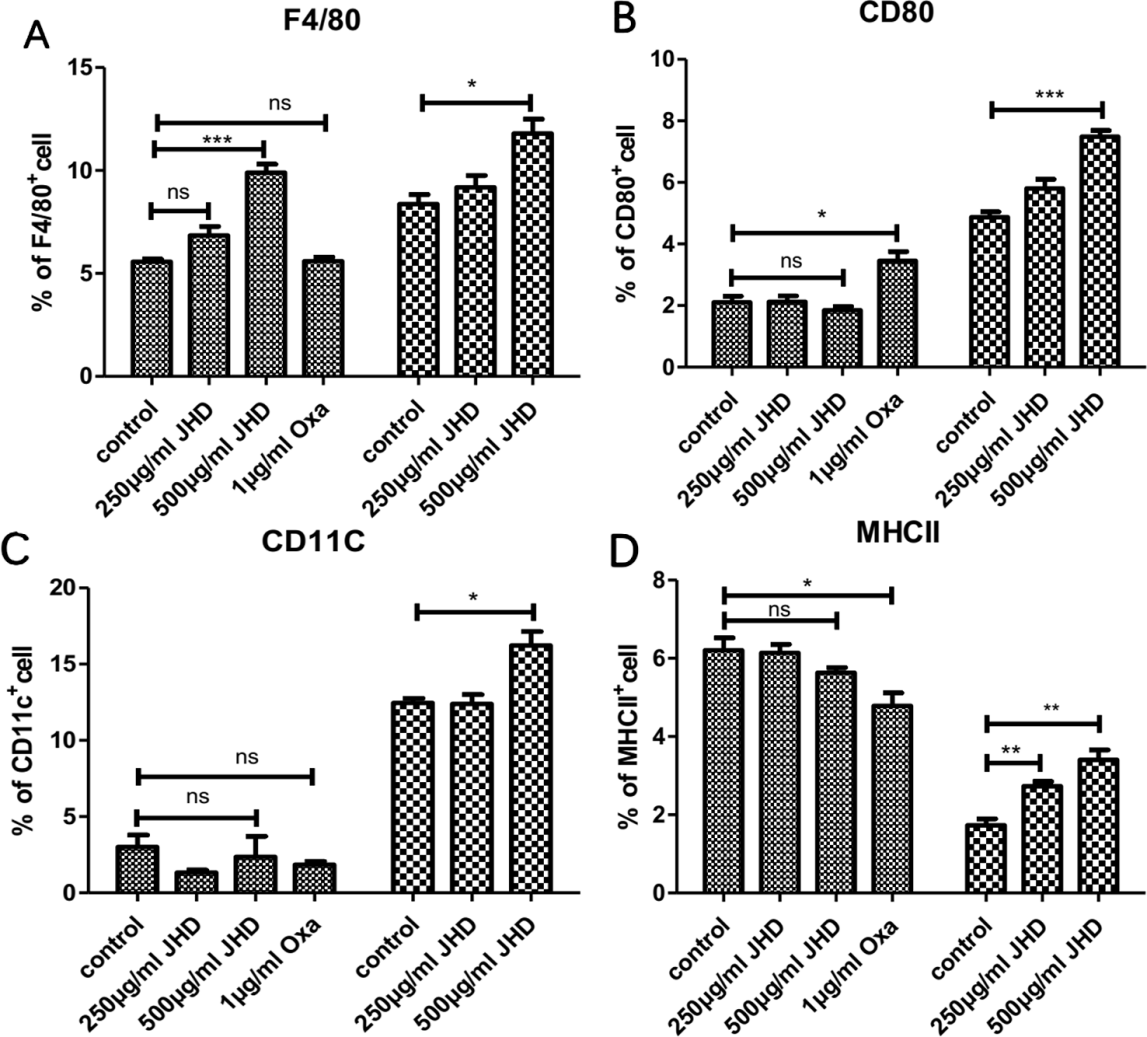
Jianpi Huayu Decoction (JHD) promotes the differentiation of Myeloid-derived suppressor cells (MDSCs) in vitro. **(A–D)** MDSCs which isolated from H_22_ hepatocellular carcinoma bearing mice by MACS technique were cultured in 24-hole culture-plate and stimulated by JHD (250μg/ml, 500μg/ml) and oxaliplatin (1μg/ml), respectively. After stimulation, cells were harvested for APC-CD11c, PE-F4/80, FITC-CD80 and Percp/Cy5.5-MHCII antibody (Biolegend) staining for flow cytometry analysis. Fc-receptor blockers were used to seal fc-receptor of cells before fluorescent antibody incubation. Statistical diagrams are shown and the left is 36 hours and the right is 48 hours, respectively (n = 3). *: *p*< 0.05; **: *p* < 0.01; ***: *p* < 0.001; ns, not significant.

As the stimulation time of JHD increased in vitro, the apoptosis of MDSCs decreased, suggesting that MDSCs might change in cell properties or type. MDSCs had been proved to be an immature heterogeneous cell population that could continue to differentiate into macrophages, dendritic cells and neutrophils. Here, MDSCs were treated with JHD and oxaliplatin in vitro, respectively. After 36h intervention with JHD (500μg/ml), the percentage of F4/80^+^cells increased (**Figure 6A**), but showed no significant difference in the expression of MHCII, CD80 and CD11c molecule (**Figures 6B–D**). Meanwhile, oxaliplatin increased expression of CD80 (**Figure 6B**) and decreased expression of MHCII (**Figure 6D**), but had no significant effect on F4/80 and CD11c molecule (**Figures 6A** and **C**). When the stimulation time of JHD was up to 48h, the percentage of CD11c^+^ and F4/80^+^ cells increased significantly (**Figures 6A** and **C**).

### JHD Weakens the Immunosuppressive Ability of MDSCs

**Figure 7:**
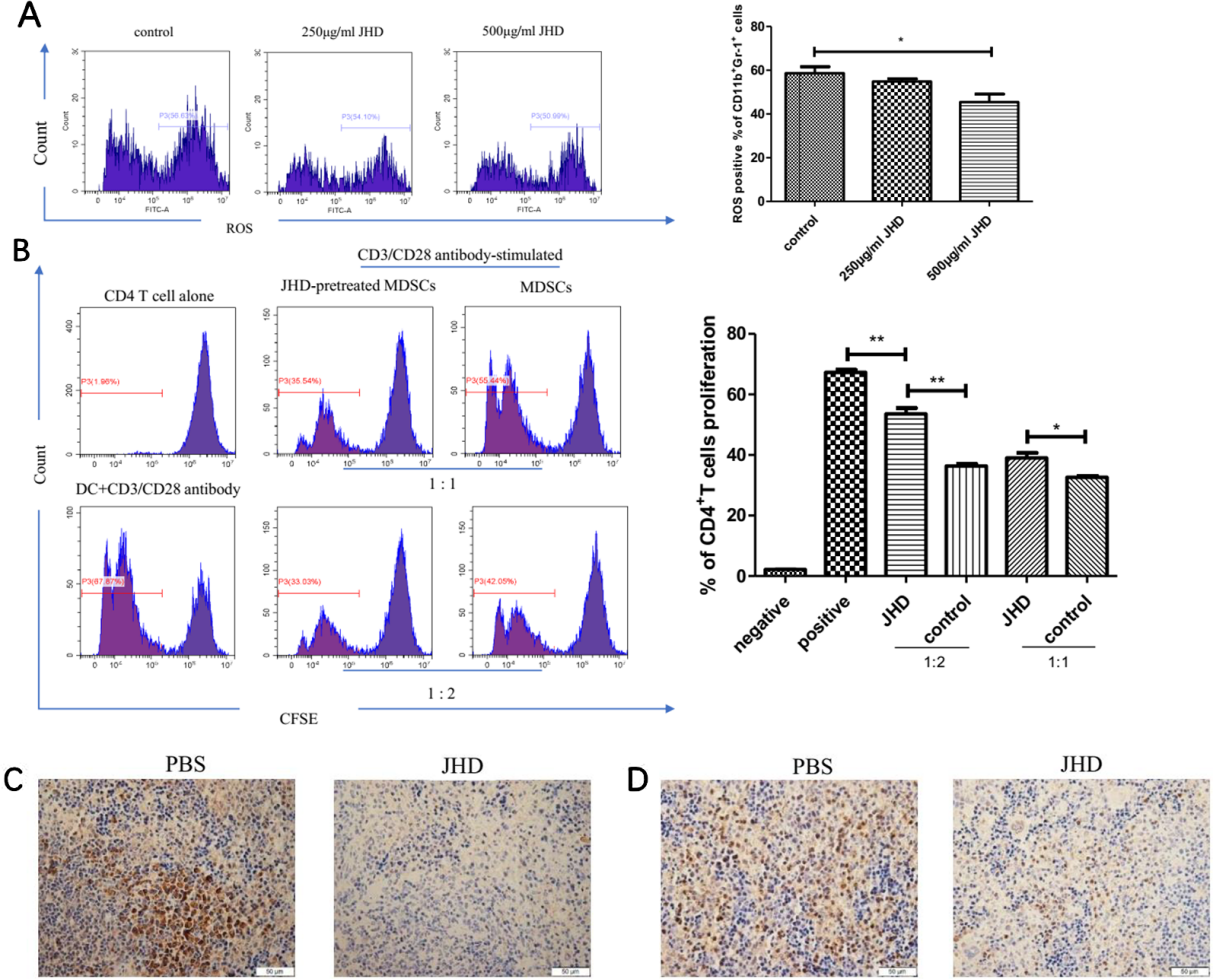
Jianpi Huayu Decoction (JHD) weakens the immunosuppressive ability of Myeloid-derived suppressor cells (MDSCs) in vitro. **(A)** Relative expression of ROS in MDSCs was analyzed by flow cytometry (n = 3). Representative flow cytometry data and statistical diagram are shown. **(B)** JHD ameliorated the proliferation inhibitive ability of MDSCs on CD4^+^ T cells. Briefly, MDSCs which isolated from H_22_ hepatocellular carcinoma bearing mice by MACS technique were pretreated with JHD (500μg/ml) or not. Naïve CD4^+^ T cells were isolated from 6-weeks BALB/c mice by Naïve CD4^+^T cells Isolation Kit, and activated by plate-coated anti‐CD3 antibody (5μg/ml, Biogems) and soluble anti‐CD28 antibody (2μg/ml, Biogems) for 12 hours. Then CD4^+^ T cells and MDSCs were co-cultured at different ratios (CD4^+^ T cells: MDSCs = 1:1, 1:2) for 48h, and CD4^+^ T cells co-cultured with DC (6 × 10^^5^ cells/well) as positive control. Proliferation of CD4^+^ T cell was measured by carboxyl fluorescein diacetate succinimide (CFSE) using flow cytometry, with the gate of CD4^+^T cells (n = 3). **(C, D)** Expression of STAT-3 and iNOS in spleen were measured by immunohistochemistry staining (×400 magnification, n = 5). Scale bar = 50μm. *: *p*< 0.05; **: *p* < 0.01; ns, not significant.

MDSCs were characterized by strong immunosuppressive and angiogenic ability. Aforesaid results suggested that JHD could change the surface molecular markers and reduced apoptosis of MDSCs, but its effect on the immunosuppressive function of MDSCs remains unclear in vitro. Here, we found that JHD could reduce expression of ROS in MDSCs after 36 hours stimulation by JHD (500μg/ml) in vitro (**Figure 7A**). Meanwhile, in vitro CD4^+^ T cells proliferative inhibition experiments showed that MDSCs pretreated with JHD (500μg/ml) had been reduced the inhibition on the proliferation of CD4^+^ T cells (**Figure 7B**). Besides, JHD could inhibit the expression of STAT-3 and iNOS in spleen (**Figures 3C, D**).

## Discussion

Hepatocellular carcinoma (HCC) is still one of deadly malignancies in human. The efficacy of transarterial chemoembolization, chemotherapy, radiotherapy and immune-checkpoint therapy for malignant HCC are still unsatisfactory (Eggert and Greten, 2017). Though the immunosuppressive environment formed by liver reduced the antigen recognition by immune cells (Jakab, 2015), but on the contrary, hindered the recognition of mutational cells. Homoplastically, tumor-induced MDSCs could significantly promote progression of tumor, by elaborately establishing immunosuppressive environment (Deng et al., 2017). According to surface markers, MDSCs in mice could be divided into the granulocytic and monocytic MDSC, both which possessed strong immunosuppressive ability (Fleming et al., 2018).

Under the condition of chronic inflammation and tumorigenesis, the differentiation of MDSCs was inhibited by a variety of factors, e.g. GM-CSF, IL-6, VEGF (Musolino et al., 2017). Pharmacological research of promoting MDSCs maturation or reducing its immunosuppressive ability was still less, although curcumin, β-glucan, all trans-retinoic acid had been studied. Traditional Chinese medicine (TCM) therapy had been confirmed as a complementary and alternative method for the treatment of various types of cancer, especially for advanced liver-cancer patient (Wang et al., 2018). JHD was a extract of herbs for oncotherapy based on TCM theoretical system and clinical practice, and here we continued to explore its mechanism based on our previous results that JHD attenuated the expression of interleukin-10 (IL-10) and transforming growth factor-beta (TGF-β) (**Supplementary Material 4**). IL-10 and TGF-β were found to be cytokines positively related to immunosuppression (Rouas et al., 2019). We speculated that JHD might attenuate HCC induced immunosuppressive microenvironment.

Firstly, we established a subcutaneous liver cancer mouse model and treated it daily with high dose of JHD (24.96g/kg) for twelve days. Results showed that JHD not only inhibited the growth of subcutaneous tumor (**Figures 2A, B**), but also reduced the structure destruction of spleen which was confirmed by H&E staining (**Figure 2F**) and significantly increased CD45.2^+^ cells in spleen (data not show). Also observed in the mouse model of H_22_ liver cancer, the splenic structure would be gradually destroyed with the progression of tumor, and that phenomenon was connected with the infiltration of MDSCs (Li et al., 2019). Combined with the above results, we considered that JHD might have regulatory effects on MDSCs. Unexpectedly, we found that the percentage of CD11b^+^Gr-1^+^ phenotypic cells and its subsets in the spleen and bone marrow of JHD-treated mice up-regulated (**Figures 3A–C**), rather than down-regulated like other studies (Nefedova et al., 2007; Kim and Kim, 2019). However, our results also demonstrated significantly increased percentage of DC (**Figure 3D**) and CTL (**Figure 4A**), and significantly decreased percentage of Th17 and Treg (**Figures 4B, C**) in spleen of JHD-treated mice. Previous studies had shown that Th17 and Treg played an important role in promoting hepatocellular carcinoma development (Gomes et al., 2016). In general, JHD could weaken the immunosuppressive status of H_22_ tumor-bearing mice, possibly by promoting MDSCs into maturation and inhibiting the immunosuppressive activity of MDSCs.

Subsequently, we further investigated the in vitro effects of JHD on HCC-induced MDSCs. Interestingly, when the concentration was lower than 250μg/ml, JHD could enhance cell viability of CD11b^+^Gr-1^+^ cells, but showed inhibitive effect when it was more than 250μg/ml dose (**Figure 5A**). After 24h stimulation, both JHD (250μg/ml, 500μg/ml) and oxaliplatin (1μg/ml) promoted apoptosis of MDSCs (**Figure 5B**). Surprisingly, at 36 hours, we observed that JHD (250μg/ml, 500μg/ml) decreased apoptosis in cultured cells, and oxaliplatin show the opposite effect (**Figure 5C**). Our previous and current studies also demonstrated that high concentrations of JHD exerted the best anti-tumor effect. Therefore, the proportion of CD11b^+^Gr-1^+^ cells in the JHD-treated group increased *in vivo*, possibly because of reduced apoptosis.

The plasticity of MDSCs and its characteristic of further differentiation into APC had been demonstrated (Tcyganov et al., 2018). In vitro, JHD significantly increased percentage of F4/80, CD11c, CD80 and MHCII positive cells in HCC-induced MDSCs, with time and concentration dependence (**Figure 5C**), which suggested that differentiation of CD11b^+^Gr-1^+^ cells were promoted by JHD. We then tested the expression of ROS in MDSCs treated with JHD or not for 36h, and found that JHD could reduce expression of ROS (**Figure 7A**). Meanwhile, we continued to co-culture CD4^+^T cells with MDSCs which were pretreated with JHD (500μg/ml) or not, and the result proved that JHD-pretreated MDSCs were weakened the proliferative inhibition ability on CD4^+^ T cells (**Figure 7B**). ROS has been proved to be a molecule that MDSCs play a role in inhibiting proliferation of CD4^+^T cells (Ohl and Tenbrock, 2018). Finally, we observed downregulated expression of both STAT-3 and inducible nitric oxide synthase (iNOS) in the spleen of JHD treated group, which was measured by immunohistochemical staining (**Figures 7C, D**). Previous studies have confirmed that STAT-3 and iNOS was related to the immunosuppressive ability of MDSCs (Diao et al., 2015; Zhao et al., 2018b).

Therefore, a comprehensive analysis of our above results suggested that increased CD11b^+^Gr-1^+^ cells in spleen of JHD-treated group appeared to have less immunosuppressive ability and could differentiate into DC. Besides, JHD could affect the apoptosis, differentiation and immunosuppressive ability of MDSCs, possibly through multiple molecule signaling pathways, e.g. NF-κB (Flores et al., 2017), STAT-3 (Guha et al., 2019), HMGB1, AMPK (Salminen et al., 2019), microRNAs (Ren et al., 2019). However, we suspected that JHD plays multiple roles by regulating STAT-3, which was involved in processes such as cell transformation, expansion and activation of MDSCs (Panni et al., 2014; Pan et al., 2016). Here, we have not yet studied the specific signaling pathways of above effect made by JHD, and based on our results, subsequent researches will be continued to explore definitely functional molecules and signaling pathways involved, to provide evidence for broadening the application of JHD.

Many antineoplastic drugs studies had shown reduced proportion of CD11b^+^Gr-1^+^ cells in tumor-bearing mice after drug treatment (Fleming et al., 2018), in addition to lenvatinib (Gunda et al., 2019) and gemcitabine (Mu et al., 2019). Frequency of CD11b^+^Gr-1^+^ cells was considered as an important factor to promote tumor progression and tumor cell resistance, and as an independent factor to evaluate the efficacy and prognosis of anti-tumor therapy (Ai et al., 2018). For comprehensive analysis, we considered that higher or lower proportion of MDSCs after antineoplastic drugs intervention still depended on the tumor type, and its direct effects on MDSCs. The increased CD11b and Gr-1 molecule did not entirely represent the immunosuppressive ability of MDSCs. Coincidentally, Safari et al. also pointed out that the proportion change of MDSCs depended on the results of systematic treatment (Safari et al., 2019).

Finally, we highlighted that JHD could weaken the immunosuppressive ability of MDSCs, promote MDSCs differentiation, increase systematic anti-tumor immune response and finally inhibited the growth of subcutaneous H_22_ hepatocellular carcinoma. Synthetically, we hold the opinion that, under condition of antineoplastic drugs administration, the increased CD11b^+^Gr-1^+^cells may not be villainy, when these drugs could suppress immunosuppressive ability of CD11b^+^Gr-1^+^cells and promote it mature to replenish dendritic cell, at the same time.

## Data Availability Statement

The datasets generated for this study are available on request to the corresponding authors.

## Ethics Statement

The animal study was reviewed and approved by The Institutional Animal Care and Use Committee of Southern Medical University.

## Author Contributions

YX, WT, and CW conceived the study protocol and assisted to coordinate arrangement of funding. YX, YR, BF, and XD performed the *in vitro* experiments and wrote this manuscript. XW and YZ carried out high performance liquid chromatography analysis of JHD. YH prepared water extract and freeze-dried powder of JHD. CZ, HW, JL, XH, and ZW participated in *in vivo* experiments. YC and XZ analyzed the data. All authors read and approved the final manuscript.

## Funding

This study was supported by the following foundation projects: The National Natural Science Foundation of China (grant NO.81774261), The Administration of Traditional Chinese Medicine of Guangdong, China (grant NO. 20191001, 20181008, 20181002, 20171001), The Natural Science Foundation of Guangdong, China (grant NO. 2018A0303130315), and The Foundation of Guangzhou Science Technology and Innovation Commission (grant NO. 201803010088, 201804010118).

## Conflict of Interest

The authors declare that the research was conducted in the absence of any commercial or financial relationships that could be construed as a potential conflict of interest.

The handling editor declared a shared affiliation, though no other collaboration, with the authors YR, BF, YH at time of review.

## References

[B1] Ai L. Mu S. Wang Y. Wang H. Cai L. Li W. (2018). Prognostic role of myeloid-derived suppressor cells in cancers: a systematic review and meta-analysis. BMC Cancer 18, 1220. 10.1186/s12885-018-5086-y 30518340 PMC6280417

[B2] Anani W. Shurin M. R. (2017). Targeting myeloid-derived suppressor cells in cancer. Adv. Exp. Med. Biol. 1036, 105–128. 10.1007/978-3-319-67577-0_8 29275468

[B3] Deng Y. Cheng J. Fu B. Liu W. Chen G. Zhang Q. (2017). Hepatic carcinoma-associated fibroblasts enhance immune suppression by facilitating the generation of myeloid-derived suppressor cells. Oncogene 36, 1090–1101. 10.1038/onc.2016.273 27593937

[B4] Diao J. Yang X. Song X. Chen S. He Y. Wang Q. (2015). Exosomal Hsp70 mediates immunosuppressive activity of the myeloid-derived suppressor cells *via* phosphorylation of Stat3. Med. Oncol. 32, 453. 10.1007/s12032-014-0453-2 25603952

[B5] Eggert T. Greten T. F. (2017). Current standard and future perspectives in non-surgical therapy for hepatocellular carcinoma. Digestion 96, 1–4. 10.1159/000464282 28605745 PMC5548590

[B6] Fleming V. Hu X. Weber R. Nagibin V. Groth C. Altevogt P. (2018). Targeting myeloid-derived suppressor cells to bypass tumor-induced immunosuppression. Front. Immunol. 9, 398. 10.3389/fimmu.2018.00398 29552012 PMC5840207

[B7] Flores R. R. Clauson C. L. Cho J. Lee B. C. McGowan S. J. Baker D. J. (2017). Expansion of myeloid-derived suppressor cells with aging in the bone marrow of mice through a NF-kappaB-dependent mechanism. Aging Cell 16, 480–487. 10.1111/acel.12571 28229533 PMC5418207

[B8] Gao J. Wang Q. Huang Y. Tang K. Yang X. Cao Z. (2019). In silico study of anti-insomnia mechanism for suanzaoren prescription. Front. Pharmacol. 10, 925. 10.3389/fphar.2019.00925 31507421 PMC6713715

[B9] Gomes A. L. Teijeiro A. Buren S. Tummala K. S. Yilmaz M. Waisman A. (2016). Metabolic inflammation-associated IL-17A causes non-alcoholic steatohepatitis and hepatocellular carcinoma. Cancer Cell 30, 161–175. 10.1016/j.ccell.2016.05.020 27411590

[B10] Guha P. Gardell J. Darpolor J. Cunetta M. Lima M. Miller G. (2019). STAT3 inhibition induces Bax-dependent apoptosis in liver tumor myeloid-derived suppressor cells. Oncogene 38, 533–548. 10.1038/s41388-018-0449-z 30158673

[B11] Gunda V. Gigliotti B. Ashry T. Ndishabandi D. McCarthy M. Zhou Z. (2019). Anti-PD-1/PD-L1 therapy augments lenvatinib's efficacy by favorably altering the immune microenvironment of murine anaplastic thyroid cancer. Int. J. Cancer 144, 2266–2278. 10.1002/ijc.32041 30515783

[B12] Jakab L. (2015). The liver and the immune system. Orv. Hetil. 156, 1203–1213. 10.1556/650.2015.30190 26186144

[B13] Kim N. R. Kim Y. J. (2019). Oxaliplatin regulates myeloid-derived suppressor cell-mediated immunosuppression *via* downregulation of nuclear factor-kappaB signaling. Cancer Med. 8, 276–288. 10.1002/cam4.1878 30592157 PMC6346236

[B14] Li B. Zhang S. Huang N. Chen H. Wang P. Yang J. (2019). CCL9/CCR1 induces myeloidderived suppressor cell recruitment to the spleen in a murine H22 orthotopic hepatoma model. Oncol. Rep. 41, 608–618. 10.3892/or.2018.6809 30365155

[B15] Lin J. Lin Y. Su L. Su Q. Guo W. Huang X. (2017). The role of Jagged1/Notch pathway-mediated angiogenesis of hepatocarcinoma cells in vitro, and the effects of the spleen-invigorating and blood stasis-removing recipe. Oncol. Lett. 14, 3616–3622. 10.3892/ol.2017.6611 28927121 PMC5588019

[B16] Mu X. Y. Wang R. J. Yao Z. X. Zheng Z. Jiang J. T. Tan M. Y. (2019). RS 504393 inhibits M-MDSCs recruiting in immune microenvironment of bladder cancer after gemcitabine treatment. Mol. Immunol. 109, 140–148. 10.1016/j.molimm.2019.02.014 30951933

[B17] Musolino C. Allegra A. Pioggia G. Gangemi S. (2017). Immature myeloid-derived suppressor cells: a bridge between inflammation and cancer (Review). Oncol. Rep. 37, 671–683. 10.3892/or.2016.5291 27922687

[B18] Nefedova Y. Fishman M. Sherman S. Wang X. Beg A. A. Gabrilovich D. I. (2007). Mechanism of all-trans retinoic acid effect on tumor-associated myeloid-derived suppressor cells. Cancer Res. 67, 11021–11028. 10.1158/0008-5472.CAN-07-2593 18006848

[B19] Ohl K. Tenbrock K. (2018). Reactive oxygen species as regulators of MDSC-mediated immune suppression. Front. Immunol. 9, 2499. 10.3389/fimmu.2018.02499 30425715 PMC6218613

[B20] Pan T. Zhong L. Wu S. Cao Y. Yang Q. Cai Z. (2016). 17beta-Oestradiol enhances the expansion and activation of myeloid-derived suppressor cells *via* signal transducer and activator of transcription (STAT)-3 signalling in human pregnancy. Clin. Exp. Immunol. 185, 86–97. 10.1111/cei.12790 26969967 PMC4908292

[B21] Panni R. Z. Sanford D. E. Belt B. A. Mitchem J. B. Worley L. A. Goetz B. D. (2014). Tumor-induced STAT3 activation in monocytic myeloid-derived suppressor cells enhances stemness and mesenchymal properties in human pancreatic cancer. Cancer Immunol. Immunother. 63, 513–528. 10.1007/s00262-014-1527-x 24652403 PMC3994288

[B22] Park S. M. Youn J. I. (2019). Role of myeloid-derived suppressor cells in immune checkpoint inhibitor therapy in cancer. Arch. Pharm. Res. 42, 560–566. 10.1007/s12272-019-01165-6 31147902

[B23] Ren W. Zhang X. Li W. Feng Q. Feng H. Tong Y. (2019). Exosomal miRNA-107 induces myeloid-derived suppressor cell expansion in gastric cancer. Cancer Manag. Res. 11, 4023–4040. 10.2147/CMAR.S198886 31190980 PMC6511657

[B24] Rivoltini L. Vernieri C. Huber V. (2019). The AURORA of a new way to value myeloid immunosuppression in cancer. Cancer Res. 79, 3169–3171. 10.1158/0008-5472.CAN-19-1081 31262832

[B25] Rouas R. Merimi M. Najar M. El Zein N. Fayyad-Kazan M. Berehab M. (2019). Human CD8(+) CD25 (+) CD127 (low) regulatory T cells: microRNA signature and impact on TGF-beta and IL-10 expression. J. Cell Physiol. 234, 17459–17472. 10.1002/jcp.28367 30805923

[B26] Safari E. Ghorghanlu S. Ahmadi-Khiavi H. Mehranfar S. Rezaei R. Motallebnezhad M. (2019). Myeloid-derived suppressor cells and tumor: current knowledge and future perspectives. J. Cell Physiol. 234, 9966–9981. 10.1002/jcp.27923 30537008

[B27] Salminen A. Kauppinen A. Kaarniranta K. (2019). AMPK activation inhibits the functions of myeloid-derived suppressor cells (MDSC): impact on cancer and aging. J. Mol. Med. (Berl.) 97, 1049–1064. 10.1007/s00109-019-01795-9 31129755 PMC6647228

[B28] Sun L. Clavijo P. E. Robbins Y. Patel P. Friedman J. Greene S. (2019). Inhibiting myeloid-derived suppressor cell trafficking enhances T cell immunotherapy. JCI Insight 4. 10.1172/jci.insight.126853 PMC6483637 30944253

[B29] Tcyganov E. Mastio J. Chen E. Gabrilovich D. I. (2018). Plasticity of myeloid-derived suppressor cells in cancer. Curr. Opin. Immunol. 51, 76–82. 10.1016/j.coi.2018.03.009 29547768 PMC5943174

[B30] Tobin R. P. Jordan K. R. Robinson W. A. Davis D. Borges V. F. Gonzalez R. (2018). Targeting myeloid-derived suppressor cells using all-trans retinoic acid in melanoma patients treated with Ipilimumab. Int. Immunopharmacol. 63, 282–291. 10.1016/j.intimp.2018.08.007 30121453 PMC6134177

[B31] Wang S. Long S. Wu W. (2018). Application of traditional Chinese medicines as personalized therapy in human cancers. Am. J. Chin. Med. 46, 953–970. 10.1142/S0192415X18500507 29986595

[B32] Wei W. Shu S. Zhu W. Xiong Y. Peng F. (2016). The kinome of edible and medicinal fungus wolfiporia cocos. Front. Microbiol. 7, 1495. 10.3389/fmicb.2016.01495 27708635 PMC5030230

[B33] Xie Y. Xiang Y. Sheng J. Zhang D. Yao X. Yang Y. (2018). Immunotherapy for hepatocellular carcinoma: current advances and future expectations. J. Immunol. Res. 2018, 8740976. 10.1155/2018/8740976 29785403 PMC5896259

[B34] Yan Z. Lai Z. Lin J. (2017). Anticancer properties of traditional chinese medicine. Comb. Chem. High Throughput Screen 20, 423–429. 10.2174/1386207320666170116141818 28093974

[B35] Yap J. Y. Wirasinha R. C. Chan A. Howard D. R. Goodnow C. C. Daley S. R. (2018). Indirect presentation in the thymus limits naive and regulatory T-cell differentiation by promoting deletion of self-reactive thymocytes. Immunology 154, 522–532. 10.1111/imm.12904 29411880 PMC6002238

[B36] Zhao Y. Shao Q. Zhu H. Xu H. Long W. Yu B. (2018a). Resveratrol ameliorates Lewis lung carcinoma-bearing mice development, decreases granulocytic myeloid-derived suppressor cell accumulation and impairs its suppressive ability. Cancer Sci. 109, 2677–2686. 10.1111/cas.13720 29959821 PMC6125446

[B37] Zhao Y. Shen X. F. Cao K. Ding J. Kang X. Guan W. X. (2018b). Dexamethasone-induced myeloid-derived suppressor cells prolong allo cardiac graft survival through inos- and glucocorticoid receptor-dependent mechanism. Front. Immunol. 9, 282. 10.3389/fimmu.2018.00282 29497426 PMC5818399

[B38] Zhao C. He R. Shen M. Zhu F. Wang M. Liu Y. (2019). PINK1/Parkin-Mediated mitophagy regulation by reactive oxygen species alleviates rocaglamide a-induced apoptosis in pancreatic cancer cells. Front. Pharmacol. 10, 968. 10.3389/fphar.2019.00968 31551778 PMC6735223

