# Supplementary material for: Jianpi Huayu Decoction Attenuates the Immunosuppressive Status of H22 Hepatocellular Carcinoma-Bearing Mice: By Targeting Myeloid-Derived Suppressor Cells
Source: Front Pharmacol. 2020 Feb 18;11:16. doi: 10.3389/fphar.2020.00016 (PMC7042893; doi:10.3389/fphar.2020.00016)
Supplement: Supplementary file 1 [file DataSheet_1.pdf]

Supplementary Material 1

A

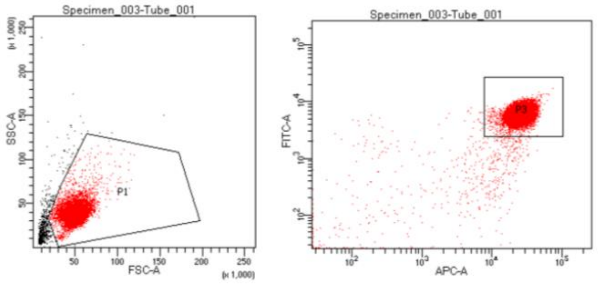

Tube: Tube_001

Population	#Events	%Parent	%Total
All Events	11,793	####	100.0
P1	11,114	94.2	94.2
P2	10,028	90.2	85.0
Q1	0	0.0	0.0
Q2	4	0.0	0.0
Q3	146	1.5	1.2
Q4	9,878	98.5	83.8
P3	10,526	94.7	89.3

B

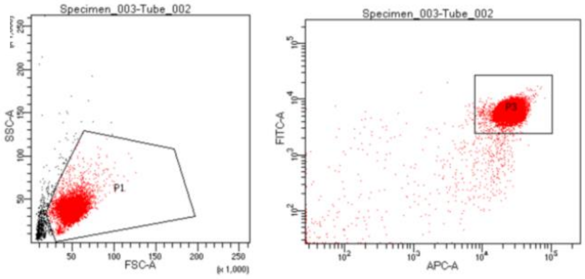

Tube: Tube_002

Population	#Events	%Parent	%Total
All Events	11,781	####	100.0
P1	11,087	94.1	94.1
P2	10,000	90.2	84.9
Q1	0	0.0	0.0
Q2	3	0.0	0.0
Q3	144	1.4	1.2
Q4	9,853	98.5	83.6
P3	10,476	94.5	88.9

C

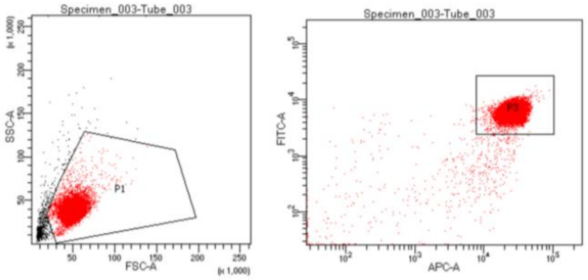

Tube: Tube_003

Population	#Events	%Parent	%Total
All Events	11,744	####	100.0
P1	11,017	93.8	93.8
P2	10,000	90.8	85.1
Q1	0	0.0	0.0
Q2	1	0.0	0.0
Q3	162	1.6	1.4
Q4	9,837	98.4	83.8
P3	10,422	94.6	88.7

Supplementary Figure 1. The purity of myeloid-derived suppressor cells which isolated from bone marrow and spleen of H22 hepatoma carcinoma-bearing mice. The purity of MDSCs was confirmed by flow cytometry (n=3) and was up to 94%.
