# Supplementary material for: Jianpi Huayu Decoction Attenuates the Immunosuppressive Status of H22 Hepatocellular Carcinoma-Bearing Mice: By Targeting Myeloid-Derived Suppressor Cells
Source: Front Pharmacol. 2020 Feb 18;11:16. doi: 10.3389/fphar.2020.00016 (PMC7042893; doi:10.3389/fphar.2020.00016)
Supplement: Supplementary file 2 [file DataSheet_2.pdf]

Supplementary Material 2

Specimen：**Radix Sophorae Flavescentis (Sophora flavescens Alt, Leguminosae)**

Batch number：181003011

Inspection item：

1. Morphological identification: meet the specification.
2. Microscopic identification：

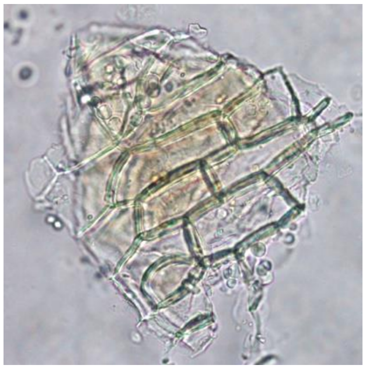
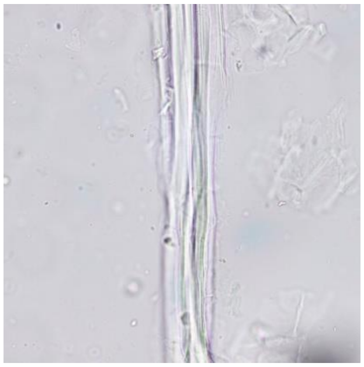
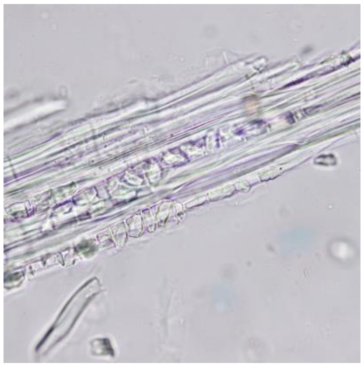

cork cell　　　　fiber　　　　crystal fiber

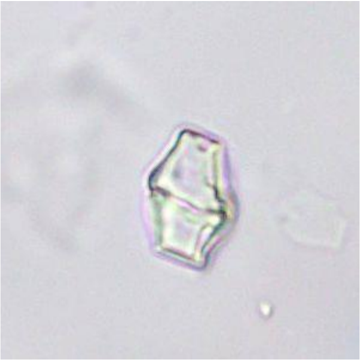
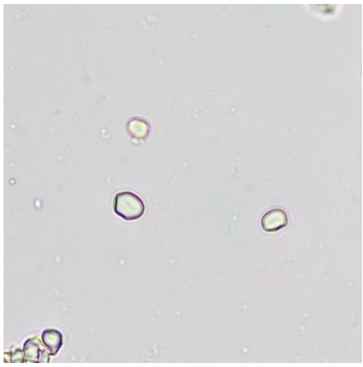
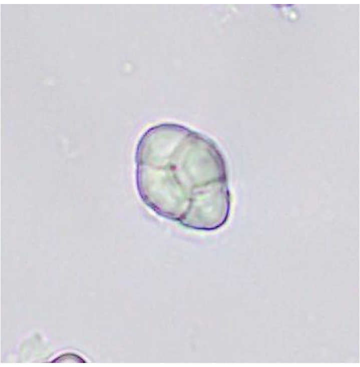

Calcium oxalate square crystal　　starch grain　　Starch granules are compound granules

3. Thin-layer chromatography：

（1）method1：

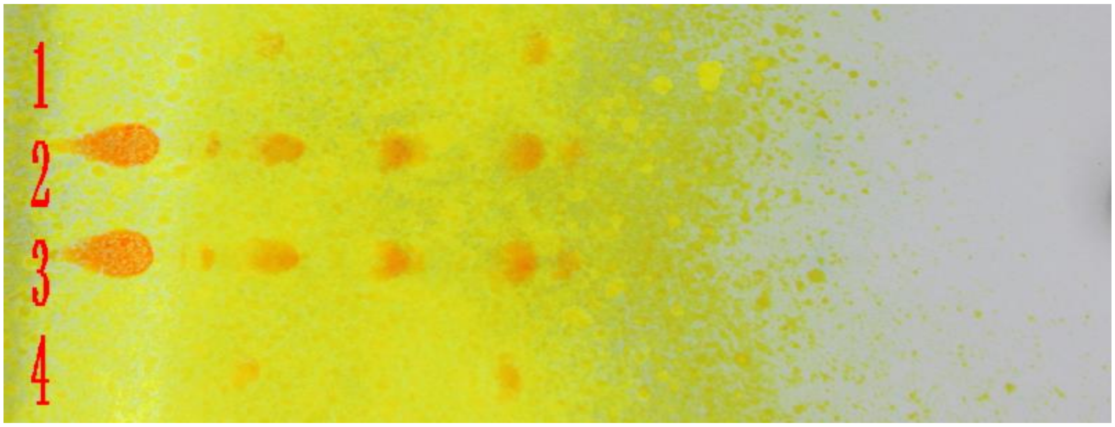

Thin layer diagram information	1,4---- Mixed solution of matrine and sophoridine	Point sample：4 μl
	2,3---- sample	Point sample：4 μl
Thin laminate information	Silica gel G thin laminate prepared by 2% sodium hydroxide solution	
Developing solvent	Toluene - acetone - methanol（8：3：0.5）+ Toluene - ethyl acetate - methanol - water（2：4：2：1）	
Chromogenic agent	Bismuth iodide potassium test solution and sodium nitrite ethanol test solution	
Viewing	white light	

（2）method 2：

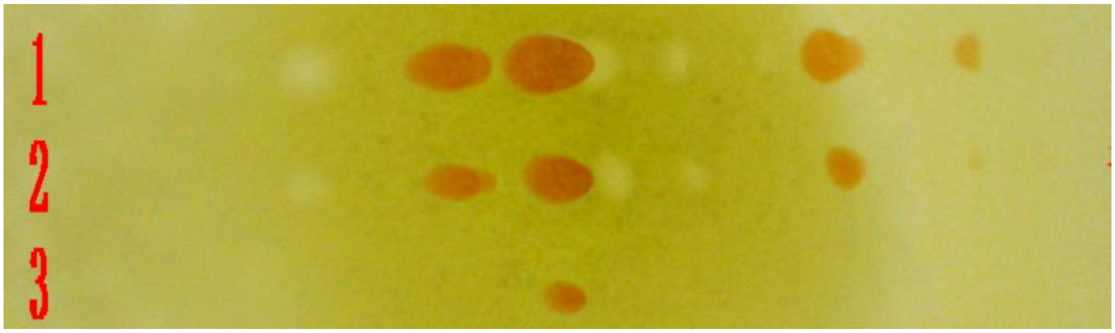

Thin layer diagram information	1 ,2---- sample	Point sample： 4 µl
	3---- oxymatrine	Point sample： 4 µl
Thin laminate information	Silica gel G thin laminate prepared by 2% sodium hydroxide solution	
Developing solvent	Trichloromethane - methanol - concentrated ammonia solution （5：0.6：0.3）	
Chromogenic agent	Bismuth iodide potassium test solution and sodium nitrite ethanol test solution	
Viewing	white light	

4. chemical identification：

- ①inspection item： Water content

  inspection provisions： < 10.5%

  result： 8.58%

- ②inspection item： total ash

  inspection provisions： < 8.0%

  result： 4.8%

③inspection item： Residual sulfur dioxide

inspection provisions： < 130mg/kg

result： 0mg/kg

- ④inspection item： Extract

inspection provisions： > 20.0%

result： 32.1%

- ⑤inspection item： content determination

inspection provisions： The total content of matrine ($C_{15}H_{24}N_2O$) and oxymatrine ($C_{15}H_{24}N_2O_2$) shall not be less than 1.3% as calculated by dry product

result： 2.2%

Conclusion: Conformed to the quality standards of Chinese pharmacopoeia.

Quality supervisor： An Luo

Specimen： **Poria (Poria cocos (Schw.) Wolf, Polyporaceae)**

Batch number： 18110671

Inspection item：

1. Morphological identification: meet the specification.
2. Microscopic identification：

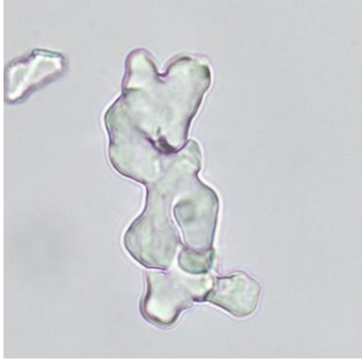

Irregular granular mass

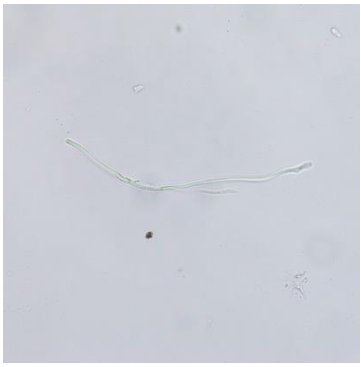

hypha

3. Thin-layer chromatography：

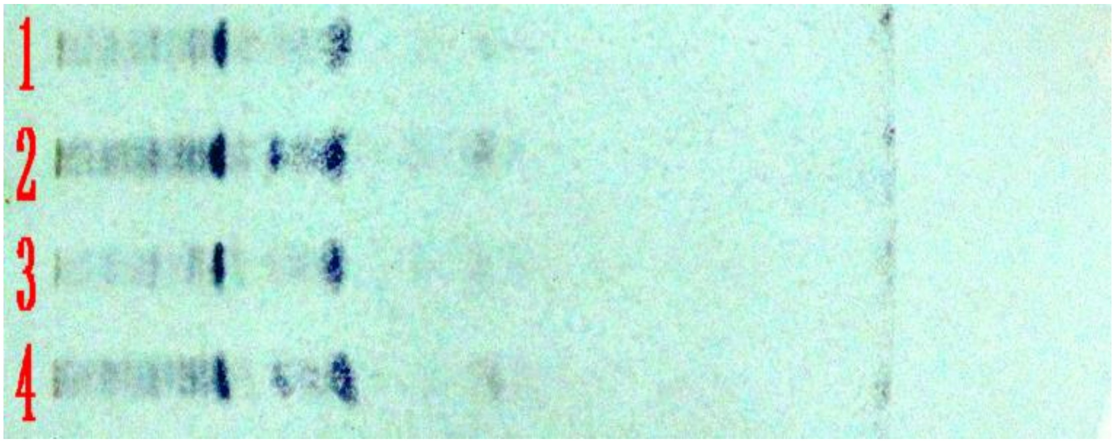

Thin layer diagram information	1,3---- Poria control herb	Point sample： 2 μl
	2,4---- sample	Point sample： 2 μl
Thin laminate information	Silicon G thin laminate	
Developing solvent	Toluene - ethyl acetate - formic acid（20:5:0.5）	
Chromogenic agent	2% Vanillin sulfuric acid solution - ethanol（4:1）	
Viewing	white light	

4. chemical identification：

- ①inspection item：Water content

  inspection provisions： < 17.5%

  result：16.1%

②inspection item：The total ash content

inspection provisions：< 2.0%

result：0.41%

③inspection item：Residual sulfur dioxide

inspection provisions：< 130mg/kg

result：0mg/kg

- ④inspection item：extractum

inspection provisions：> 3.0%

result：4.8%

Conclusion: Conformed to the quality standards of Chinese pharmacopoeia.

Quality supervisor：An Luo

Specimen：**Fructus Citri Sarcodactylis (Citrus medica Linn. var. sarcodactylis (Noot.) Swingle, Rutaceae)**

Batch number：180902031

Inspection item：

1. Morphological identification: meet the specification.
2. Microscopic identification：

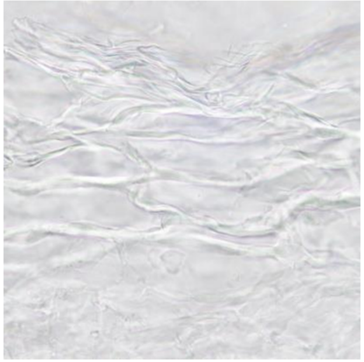
Pericarp parenchyma

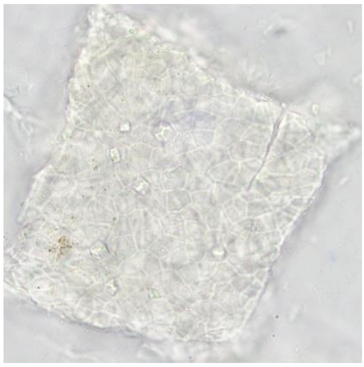
Surface appearance of epidermal cells in pericarp

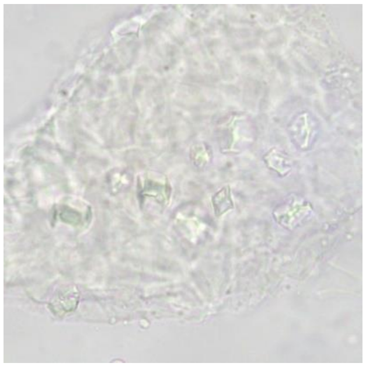
Calcium oxalate square crystal

3. thin-layer chromatography：

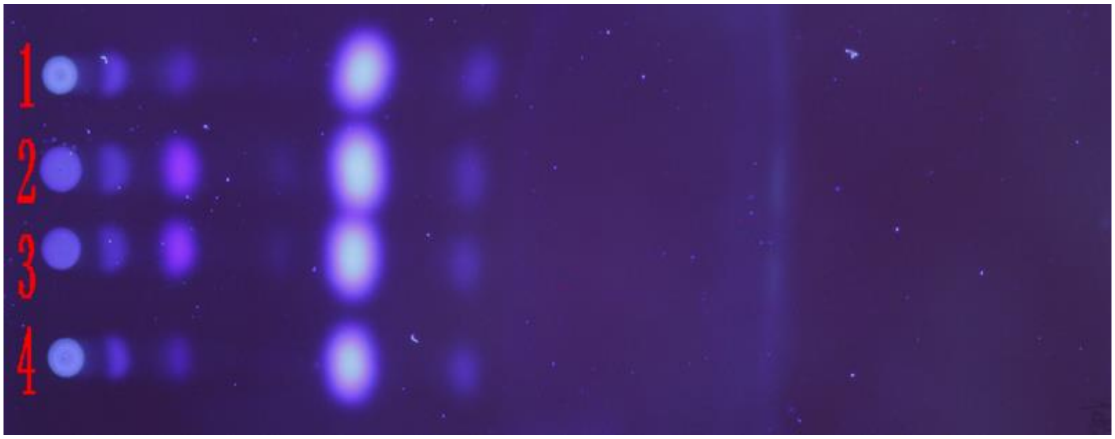

Thin layer diagram information	1,4---- Fructus Citri Sarcodactylis control herbs	Point sample： 2 μl
	2,3---- sample	Point sample： 2 μl

Thin laminate information	Silicon G thin laminate
Developing solvent	Ethyl cyclohexane-acetate（3：1）
Chromogenic agent	/
Viewing	UV365nm

3. chemical identification：

- ①inspection item：Water content

  inspection provisions：< 14.5%

  result：13.3%

  ②inspection item：Residual sulfur dioxide

  inspection provisions：< 130mg/kg

  result：0mg/kg

- ③inspection item：extractum

  inspection provisions：> 10.0%

  result：33.3%

- ④inspection item：content determination

  inspection provisions：For dried products, the content of hesperidin (C28H34O15) shall not be less than 0.030%.

  result：0.037%

Conclusion: Conformed to the quality standards of Chinese pharmacopoeia.

Quality supervisor：An Luo

Specimen：**Rhizoma Curcumae (Curcuma zedoaria (Christm.) Rosc, Zingiberaceae)**

Batch number：181202941

Inspection item：

1. Morphological identification: meet the specification.
2. Microscopic identification：

- （1）Rhizome transection：

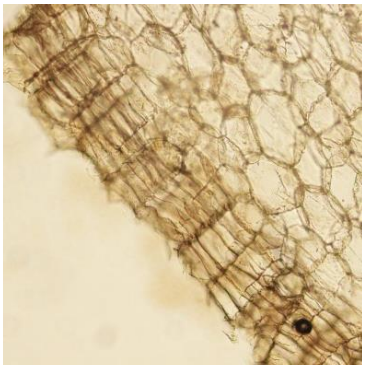
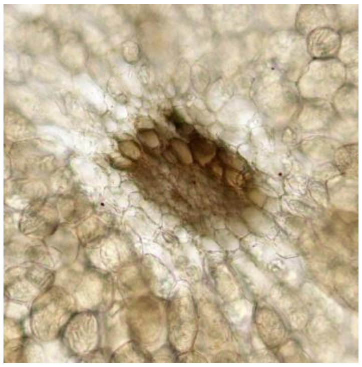
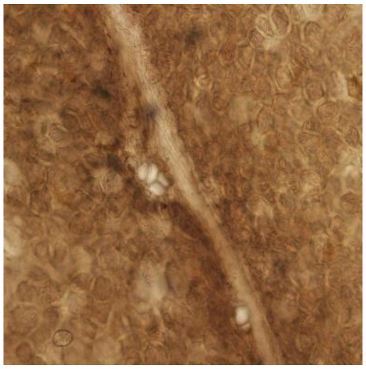

suberized cell　　Cortical trace vascular bundles　　endothelial cells

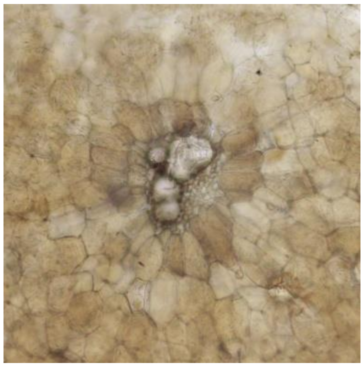
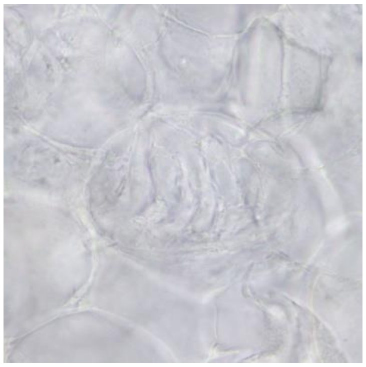

Central column external tough vascular bundl

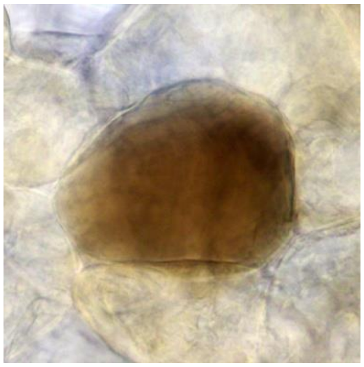
cell containing a golden, oily ubstance

（2）powder：

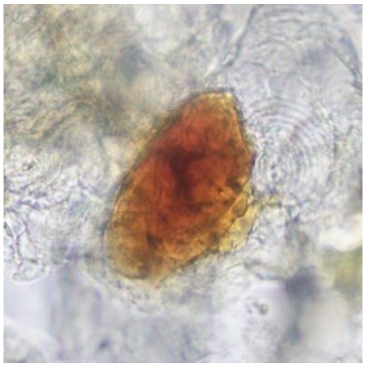
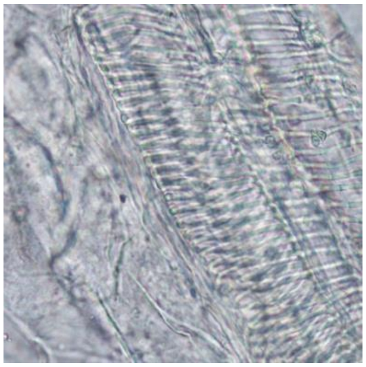
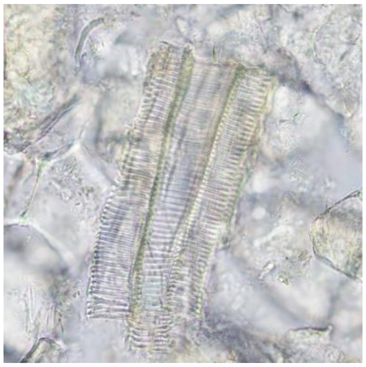

eleocyte spiral duct scalariform vessel

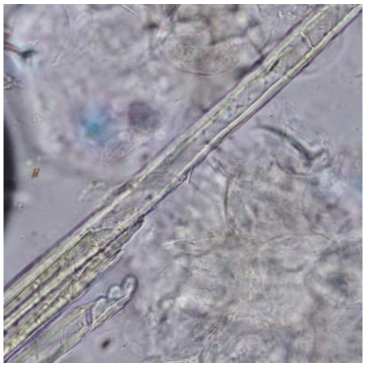
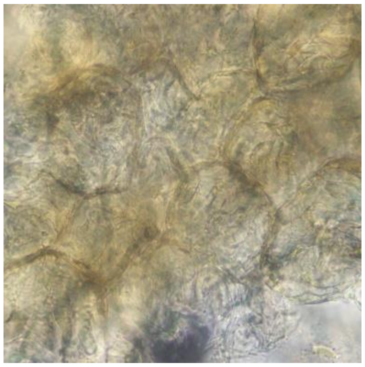

fiber Starch grains are mostly gelatinized

3. Thin-layer chromatography：

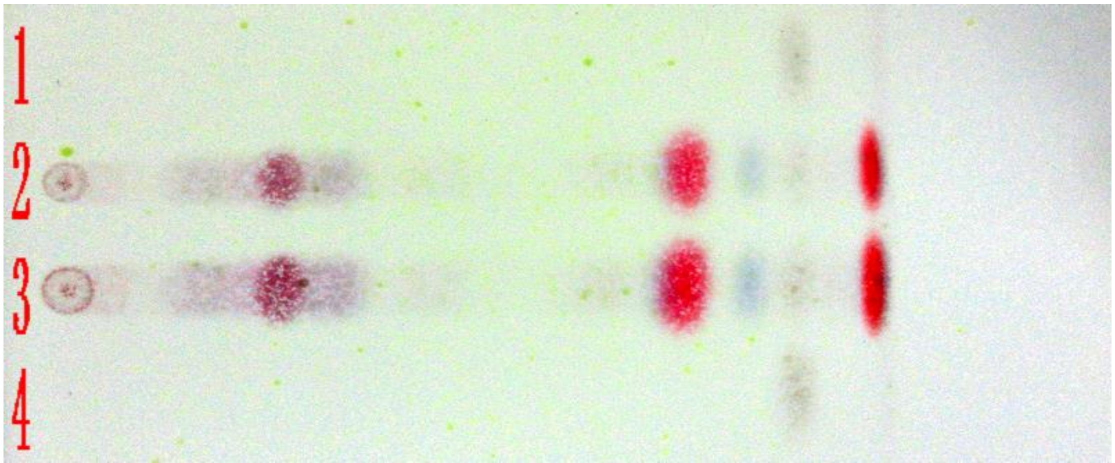

Thin layer diagram information	1,4----Germacrone	Point sample： 10 μl
	2,3---- sample	Point sample： 10 μl
Thin laminate information	Silicon G thin laminate	
Developing solvent	Petroleum ether（30～60℃）- Acetone - ethyl acetate（94:5:1）	
Chromogenic agent	1% Vanillin sulfuric acid solution	
Viewing	white light	

•

4. Chemical identification：

①inspection item： absorbance

inspection provisions： > 0.45

result： 1.1

• ②inspection item： Water content

inspection provisions：< 13.5%

result：10.2%

- ③inspection item：total ash

inspection provisions：< 7.0%

result：4.0%

④inspection item：acid-insoluble ash

inspection provisions：< 2.0%

result：1.2%

⑤inspection item：Residual sulfur dioxide

inspection provisions：< 130mg/kg

result：0mg/kg

⑥inspection item：extractum

inspection provisions：> 7.0%

result：13%

- ⑦inspection item：content determination

inspection provisions：Contain not less than 1.6% volatile oil（ml/g）

result：1.7%（ml/g）

Conclusion: Conformed to the quality standards of Chinese pharmacopoeia.

Quality supervisor：An Luo

Specimen：**Rhizoma Atractylodis Macrocephalae (Atractylodes macrocephala Koidz, Compositae)**

Batch number：181003941

Inspection item:

1. Morphological identification: meet the specification.
2. Microscopic identification：

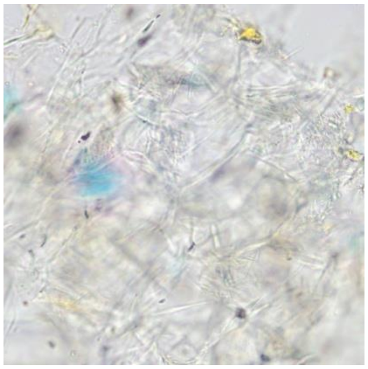

calcium oxalate crystal

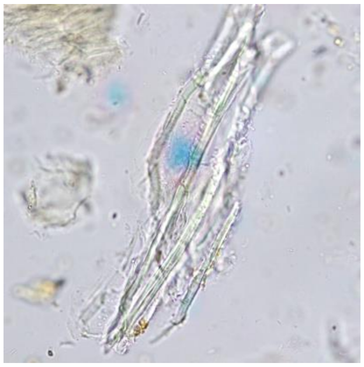

fiber

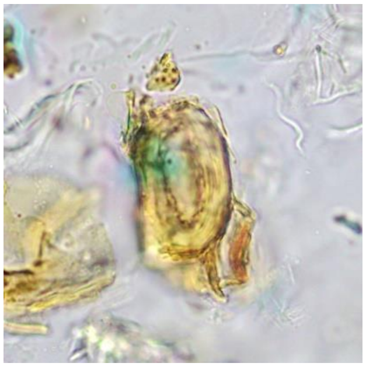

stone cell

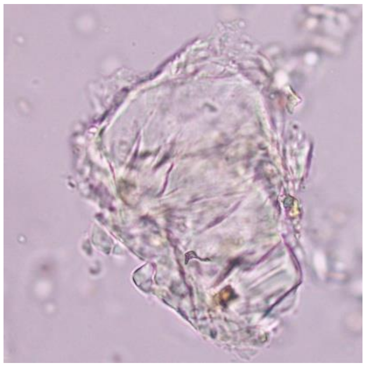

synanthrin

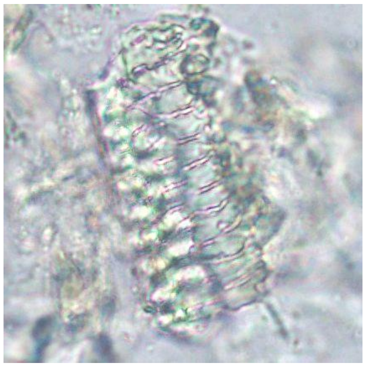

reticulate vessel

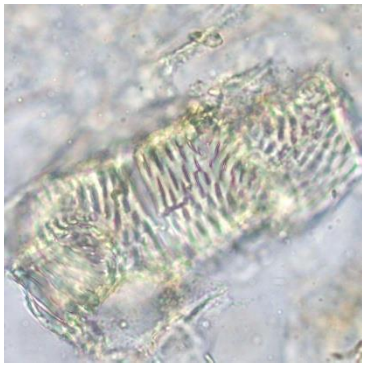

bordered pit vessel

3. Thin-layer chromatography：

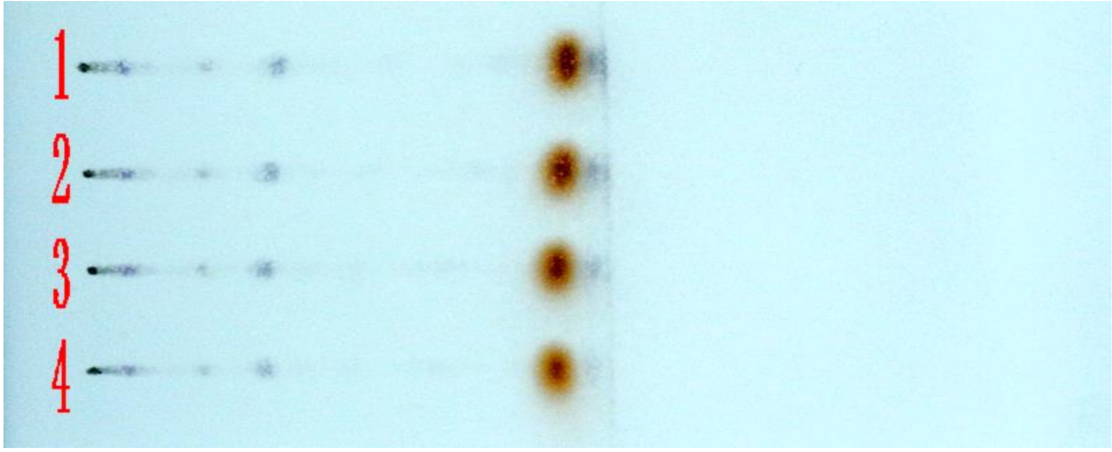

Thin layer diagram information	1, 4---- Rhizoma Atractylodis Macrocephalae control herbs	Point sample： 10 μl
	2, 3----sample	Point sample： 10 μl
Thin laminate information	Silicon G thin laminate	
Developing solvent	petroleum ether（60～90℃） - ethyl acetate（50：1）	
Chromogenic agent	5% Vanillin sulfuric acid solution	
Viewing	white light	

4. Chemical identification：

①inspection item： Water content

inspection provisions： < 14.5%

result： 13.6%

②inspection item：The total ash content

inspection provisions：< 5.0%

inspection result：3.6%

③inspection item：Residual sulfur dioxide

inspection provisions：< 350mg/kg

inspection result：248 mg/kg

④inspection item：chromaticity

inspection provisions： According to the solution color test method, compared with the yellow no. 9 standard color solution, should not be deeper.

inspection result：meet the specification

⑤inspection item：extractum

inspection provisions：> 35.0%

inspection result：39.8%

Conclusion: Conformed to the quality standards of Chinese pharmacopoeia.

Quality supervisor：An Luo

Specimen: **Hedyotis diffusa (Hedyotis diffusa Willd. Var. Longipes Nakai, Rubiaceae)**

Batch number: 181103361

Inspection item：

1. Morphological identification: meet the specification.
2. Thin-layer chromatography：

- 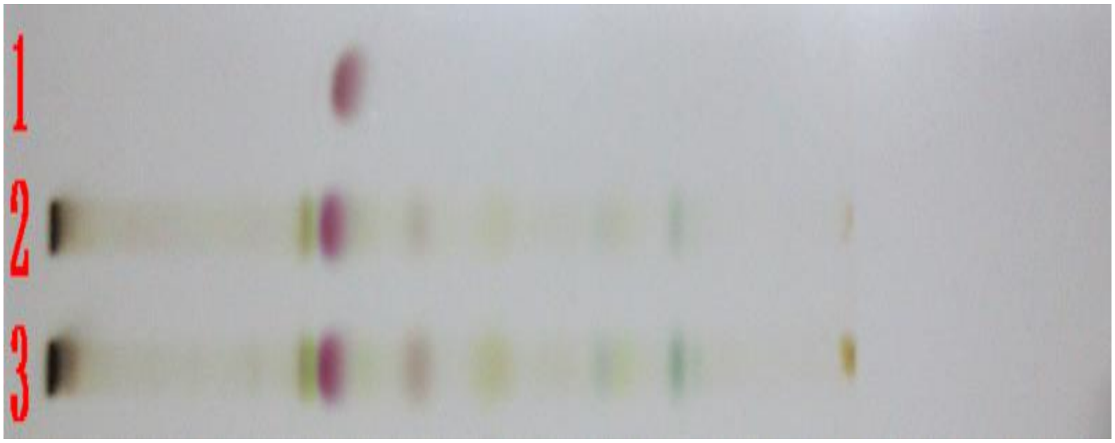

Thin layer diagram information	1---- Oleanolic acid reference	Point sample：__5__ μl
	2---- Hedyotis diffusa reference	Point sample：__5__ μl
	3---- sample	Point sample：__5__ μl
Thin laminate information	Silicon G thin laminate	
Developing solvent	petroleum ether（30～6 0℃) - Toluene - ethyl acetate - glacial acetic acid（20:40:14:1）	
Chromogenic agent	10% Sulfuric acid ethanol solution	
Viewing	white light	

3. Chemical identification：

①inspection item: Water content

inspection provisions：<12.5%

Result：10.1%

②inspection item: Residual sulfur dioxide

inspection provisions：< 130mg/kg

Result：0mg/kg

③inspection item: Extractum

inspection provisions：> 6.0%

inspection result：9.8%

④inspection item: Content determination

inspection provisions： For dry products, the total amount of Oleanolic acid (C30H48O3) and Ursolic acid (C30H48O3) shall not be less than 0.15%.

Result：0.037%

Conclusion: Conformed to the quality standards of Chinese pharmacopoeia.

Quality supervisor：An Luo
