# Supplementary material for: Jianpi Huayu Decoction Attenuates the Immunosuppressive Status of H22 Hepatocellular Carcinoma-Bearing Mice: By Targeting Myeloid-Derived Suppressor Cells
Source: Front Pharmacol. 2020 Feb 18;11:16. doi: 10.3389/fphar.2020.00016 (PMC7042893; doi:10.3389/fphar.2020.00016)
Supplement: Supplementary file 3 [file DataSheet_3.pdf]

Supplementary Material 3

Abstract

We had completed the qualitative and quantitative analysis of Jianpi Huayu Decoction, by using high-performance liquid chromatography with diode array detection (HPLC-DAD) method. Here, Methodology analysis included Linear relation, stability, repeatability, precision and recovery test. The results demonstrated that JHD had good stability and repeatability, which could be used for the follow-up in vivo and in vitro studies.

1 Materials and methods

1.1 Materials

1.2 Preparation of Jianpi Huayu Decoction Extract

The herbs of JHD were consisted of Fructus Citri Sarcodactylis (Citrus medica Linn. var. sarcodactylis (Noot.) Swingle, Rutaceae) 12g, Rhizoma Curcumae (Curcuma zedoaria (Christm.) Rosc, Zingiberaceae) 12g, Rhizoma Atractylodis Macrocephalae (Atractylodes macrocephala Koidz, Compositae) 12g, Radix Sophorae Flavescentis (Sophora flavescens Alt, Leguminosae) 20g, Poria (Poria cocos (Schw.) Wolf, Polyporaceae) 20g, and Hedyotis diffusa (Hedyotis diffusa Willd. Var. Longipes Nakai, Rubiaceae) 20g, and purchased from Guangdong Provincial People's Hospital (Guangzhou, China). To prepare water extract of JHD, all herbs were blended into double-distilled water (1:10, Weight/Volume) for 30min and then heated for 1hour. Then water extracts were evaporated to 4g/ml herb water extract. Partial water extract was processed into lyophilized powder. All extract was stored at -80℃ until used.

1.3 Instrument and reagents

Agilent 1260 HPLC system (Agilent Technologies), Ultrasonic cleaner (Autoscience) , Rotary evaporators, Chromatographic column: Agilent ZORBAX SB-C18 column (4.6 ×250 mm, 5 μm); Hesperidin (HPLC, >98% purity), p-Coumaric Acid (HPLC, >98% purity), Ferulic Acid (HPLC, >98% purity), 5,7-Dimethoxycoumarin (HPLC, >98% purity) and Bergapten (HPLC, >98% purity) were purchased from Chengdu Chroma-Biotechnology Co., Ltd (Chengdu, China)； Chromatographic methanol (Merck).

1.2 Methods

1.2.1 Preparation of reference solution and sample solution

p-Coumaric Acid, Ferulic Acid, Hesperidin, 5,7-Dimethoxycoumarin and Bergapten were accurately weighed, placed in 10mL volumetric flask, dissolved with methanol and diluted to the scale, to prepare the solution of p-Coumaric Acid, Ferulic Acid, Hesperidin, 5,7-Dimethoxycoumarin and Bergapten, store it at 4℃ for subsequent use, and dilute it to the required concentration. Accurately weigh 20mg of JHD, place it in 10mL volumetric flask, dissolve it with methanol and dilute it to the scale, shake it well, prepare the solution of JHD, store it at 4℃for subsequent use, and dilute it to the

required concentration. When used, the sample solution was filtered through 0.22μm microporous filter membrane.

1.2.2 Chromatographic condition of HPLC

High-performance liquid chromatography (HPLC) analysis was performed by Agilent 1260 HPLC system (Agilent Technologies). The mobile phase system was composed of water with 0.05% phosphoric acid (A) and methanol (B). The gradient elution profile was: 0～20min, 5%～21% B; 20～55 min, 21%～88% B; 55～65 min; 88% B. JHD specimens were separated by Agilent ZORBAX SB-C18 column (4.6 ×250 mm, 5 μm) at 30°C, with 1.0 ml/min of mobile phase flow rate. The UV spectrum was set at 283nm. The reference substances were detected under the same condition of JHD.

2 Results

2.1 Qualitative analysis of methanol extract from JHD

Under optimal chromatographic conditions, the chromatogram of p-Coumaric Acid (CAS: 501-98-4), Ferulic Acid (CAS:1135-24-6), Hesperidin (CAS:520-26-3), 5,7-Dimethoxycoumarin (CAS: 487-06-9) and Bergapten (CAS: 484-20-8) were shown in Figure 1A. The chromatogram of JHD methanol extract was shown in Figure 2B. The retention time of p-Coumaric Acid, Ferulic Acid, Hesperidin, 5,7-Dimethoxycoumarin and Bergapten were 37.164min, 38.096min, 39.496min, 48.149min, 49.529min, respectively.

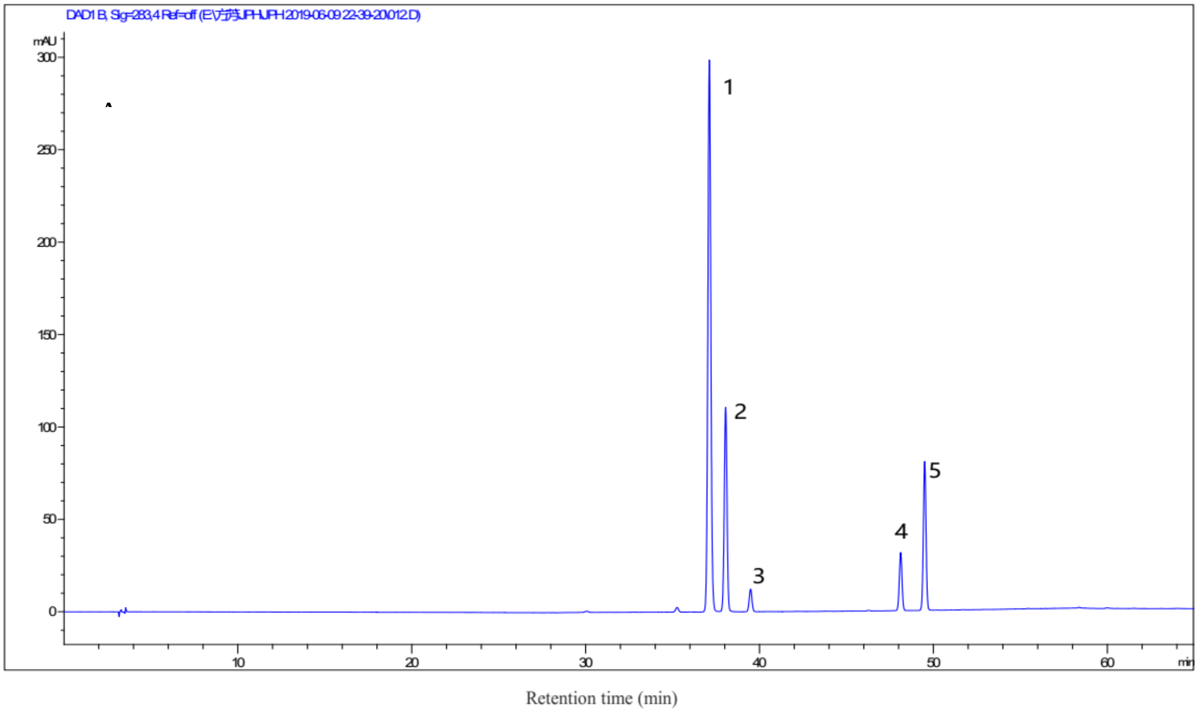

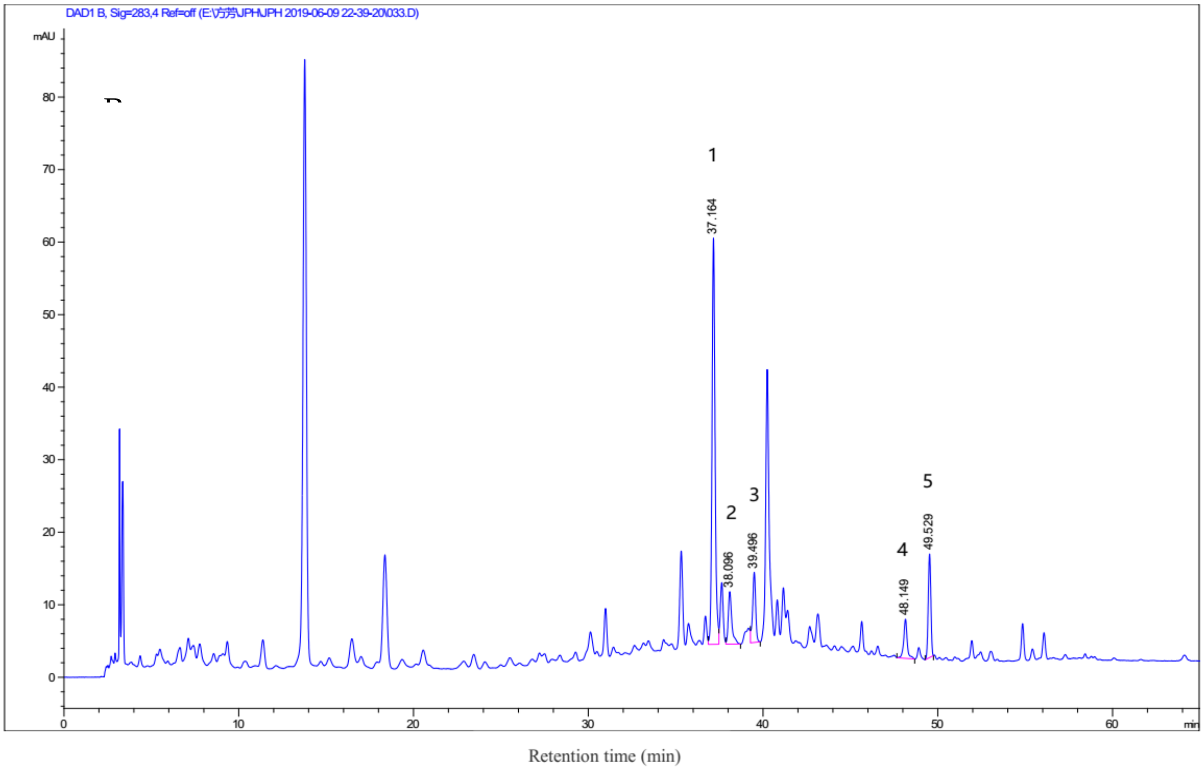

Figure 1.

Qualitative analysis of methanol extract from JHD and five reference substance. 1. p-Coumaric Acid; 2. Ferulic Acid; 3. Hesperidin; 4. 5,7-Dimethoxycoumarin; 5. Bergapten.

2.2 Method validation

2.2.1 Linear relation

Precise absorption of 100μl of the mixed solution of reference substance to 10 ml volume was diluted with methanol to the scale. 10μl of the mixed solution of reference substance was taken and the sample was injected for accurate analysis. The peak area A (Y-axis) was determined, and the linear regression of mass concentration C (X-axis) was carried out with peak area A by external standard method. Equation of linear regression and correlation coefficient (R2) of Hesperidin, p-Coumaric Acid, Ferulic Acid, 5,7-Dimethoxycoumarin and Bergapten were shown in Figure 2. There was a good linear relationship in a certain concentration range(R2>0.999).

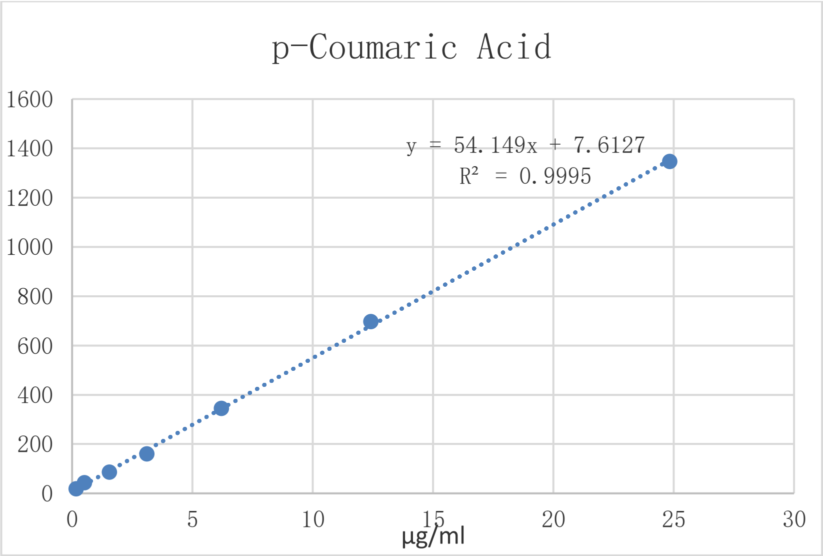
p-Coumaric Acid
y = 54.149x + 7.6127
R² = 0.9995
1600
1400
1200
1000
800
600
400
200
0
0
5
10
15
20
25
30
μg/ml

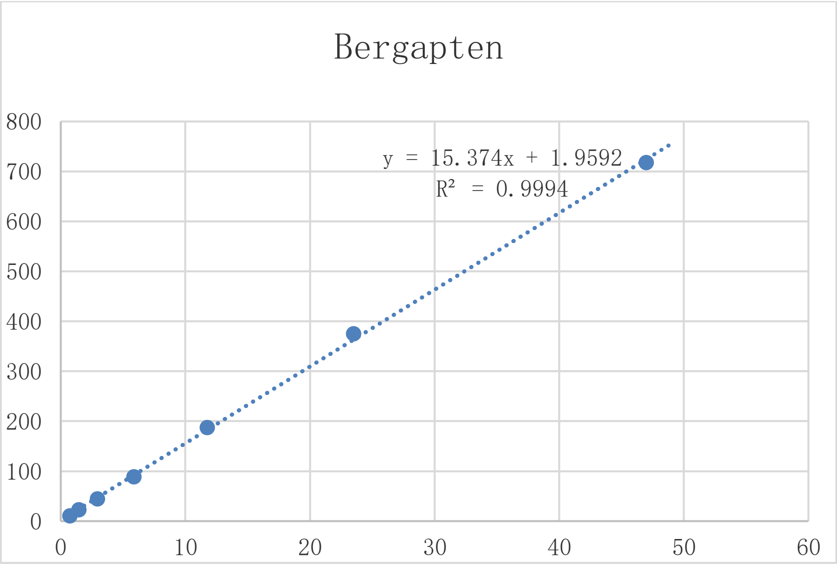
Bergapten
y = 15.374x + 1.9592
R² = 0.9994
800
700
600
500
400
300
200
100
0
0
10
20
30
40
50
60

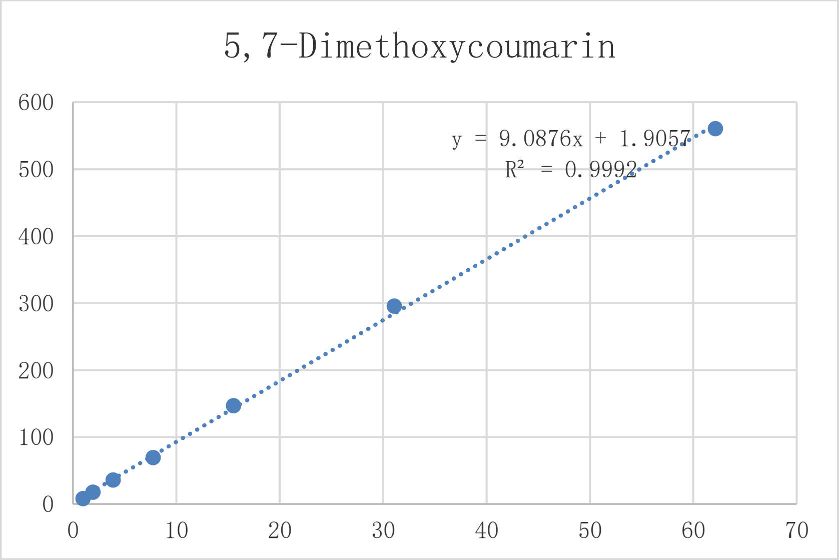
5,7-Dimethoxycoumarin
y = 9.0876x + 1.9057
R² = 0.9992
600
500
400
300
200
100
0
0
10
20
30
40
50
60
70

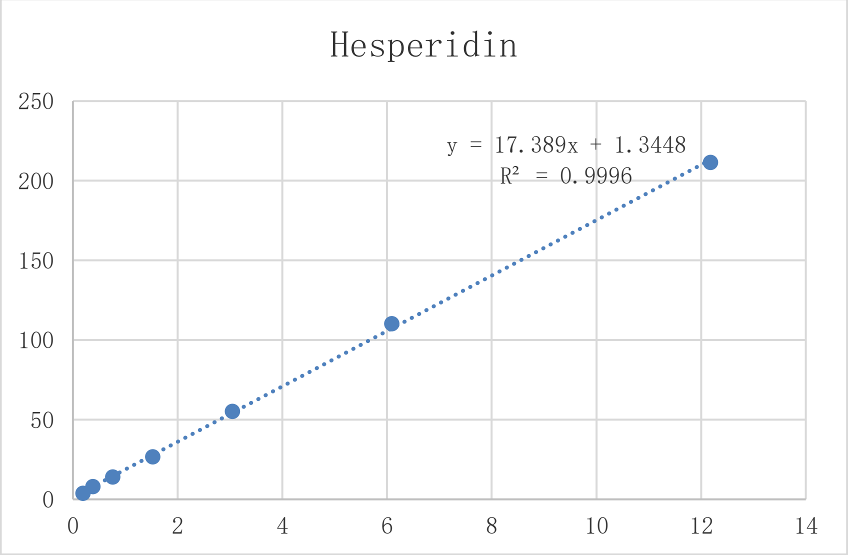
Hesperidin
y = 17.389x + 1.3448
R² = 0.9996
250
200
150
100
50
0
0
2
4
6
8
10
12
14

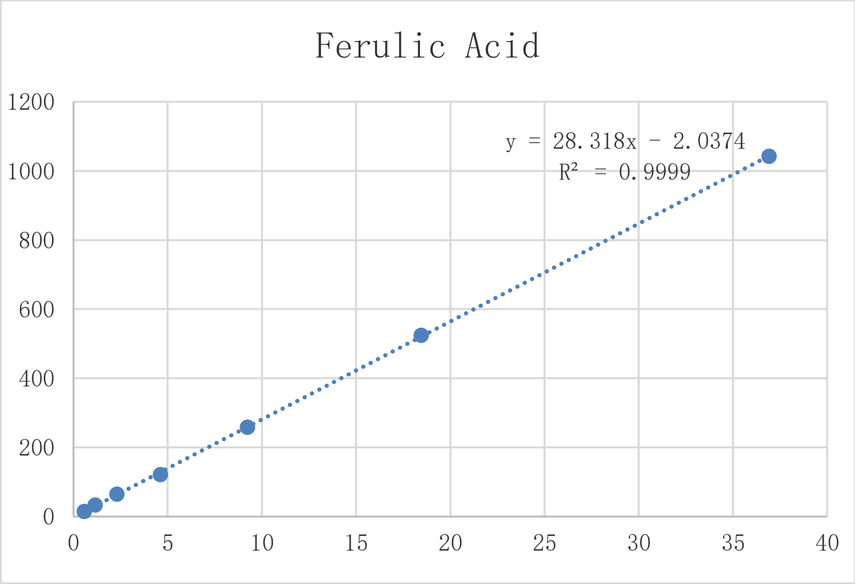

Figure 2. Linear relation of Hesperidin, p-Coumaric Acid, Ferulic Acid, 5,7-Dimethoxycoumarin and Bergapten.

2.2.2 Precision

The sample solutions were accurately absorbed with 10μl, and the samples were repeated five times for determination. Relative standard deviation (RSD) of p-Coumaric Acid, Ferulic Acid, Hesperidin, 5,7-Dimethoxycoumarin and Bergapten were 1.649%, 1.521%, 1.342%, 0.924% and 0.837%, respectively. The results showed that it had good precision, shown in Table 1.

Compound	Peak area					RSD (%)
p-Coumaric Acid	1441.4	1386.4	1386.9	1392	1397.8	1.649
Ferulic Acid	1117.2	1078.3	1078.2	1081	1085.9	1.521
Hesperidin	223	216.9	216	216	218	1.342
5,7-Dimethoxycoumarin	604.9	592.1	592	592.8	596.9	0.924
Bergapten	767.2	752.1	752.1	755.2	759.4	0.837

Table 1. Precision test.

2.2.3 Repeatability

A total of six JHD samples of the same quality were accurately weighed and determined under the above chromatographic conditions, after preparation of the sample solution in accordance with operation 1.2.2. Relative standard deviation (RSD) of Peak area of p-Coumaric Acid, Ferulic Acid, Hesperidin, 5,7-Dimethoxycoumarin and Bergapten were 1.654%, 2.338%, 2.498%, 1.883% and 2.392%, respectively. The above data indicated that the method had good reproducibility, shown in Table 2.

Compound	Peak area						RSD (%)
p-Coumaric Acid	678.7	683.1	667.6	656.3	665.8	684.2	1.654
Ferulic Acid	92.3	92.2	94	95.3	95.6	98	2.338
Hesperidin	110.4	111.6	117.3	111	116	112.9	2.498
5,7-Dimethoxycoumarin	73.9	73.6	75.2	76.9	75	73	1.883
Bergapten	149	147.9	157.5	150.9	147.9	151.1	2.392

Table 2. Repeatability test.

2.2.4 Stability

Under the above chromatographic conditions, the sample solutions were examined every 2 hours within 12 hours, and were determined 6 times in parallel. Relative standard deviation (RSD) of Peak area of p-Coumaric Acid, Ferulic Acid, Hesperidin, 5,7-Dimethoxycoumarin and Bergapten were 1.115%, 2.186%, 0.636%, 1.024% and 1.649%, respectively. The results showed that p-Coumaric Acid, Ferulic Acid, Hesperidin, 5,7-Dimethoxycoumarin and Bergapten remained stable within 12 hours, shown in Table 3.

Compound	Peak area						RSD (%)
p-Coumaric Acid	667.2	665.9	667.6	676.2	665.8	684.2	1.115
Ferulic Acid	97.2	95.5	97	95.8	95.6	101.1	2.186

Hesperidin	117.8	116.9	117.3	116.5	116	117.9	0.636
5,7-Dimethoxycoumarin	74.1	73.2	75.2	74.8	75	75	1.024
Bergapten	157.6	157.3	157.5	158.9	163.9	157.1	1.649

Table 3. Stability test.

2.2.5 Recovery

A recovery test was carried out to evaluate the accuracy of the analytical method. A total of 5 samples (0.3g/sample) were accurately weighed, each of which was added to 1mL standard solution for determination. Average recovery rate of p-Coumaric Acid, Hesperidin, 5,7-Dimethoxycoumarin and Bergapten were 97.58%, 105.66%, 96.66%, 97.31%, respectively. Relative standard deviation (RSD) of Peak area of p-Coumaric Acid, Ferulic Acid, Hesperidin, 5,7-Dimethoxycoumarin and Bergapten were 1.926%, 0.685%, 1.594% and 1.963%, respectively.

3 Concentration determination

By using the above detection method, we determined the concentrations of Hesperidin, p-Coumaric Acid, Ferulic Acid, 5,7-Dimethoxycoumarin and Bergapten in the samples, and the results were 341.3894μg/g, 602.3419μg/g, 187.500μg/g, 433.4916μg/g, 555.2405μg/g, respectively.

4 Conclusion

The content and concentration of Hesperidin, p-Coumaric Acid, Ferulic Acid, 5,7-Dimethoxycoumarin and Bergapten in JHD was determined by HPLC. And the methodological method has good stability, precision and reproducibility.
