# Supplementary material for: Jianpi Huayu Decoction Attenuates the Immunosuppressive Status of H22 Hepatocellular Carcinoma-Bearing Mice: By Targeting Myeloid-Derived Suppressor Cells
Source: Front Pharmacol. 2020 Feb 18;11:16. doi: 10.3389/fphar.2020.00016 (PMC7042893; doi:10.3389/fphar.2020.00016)
Supplement: Supplementary file 4 [file DataSheet_4.pdf]

Supplementary Material 4

Published paper

实验与应用研究

现代医院 2014 年 9 月第 14 卷第 9 期 专业技术篇 Modern Hospital Sept 2014 Vol 14 No 9

健脾化瘀方促进小鼠肝癌休眠的作用及其免疫机制研究

黄旭晖　张晓文　梁荣华　郭　苇　陈燕銮　王昌俊

JIANPI HUAYU FORMULA ON THE PROMOTION OF TUMOR DORMANCY AND THE IMMUNE MECHANISM IN MICE WITH HEPATIC CARCINOMA

HUANG Xuhui，ZHANG Xiaowen，LIANG Ronghua，et al

【摘　要】 目的　通过建立小鼠肝癌休眠模型，并予健脾化瘀法干预，研究其促进肿瘤休眠的作用及其免疫机制。方法　建立小鼠肝癌休眠模型。并随机分为 4 组：空白对照组、化疗对照组、中药低剂量组、中药高剂量组，分别给予生理盐水、卡莫氟、中剂量健脾化瘀方及高剂量健脾化瘀方进行干预。处死小鼠后，接种部位取材，免疫组化染色检测 Ki－67、TGF－β、IL－10 等相关指标的表达。结果　①肿瘤组织休眠情况：在反复外伤刺激下，空白对照组休眠率为 0。化疗对照组休眠率为 80.0%。中药低剂量组和高剂量组休眠率均为 86.7%；②对 Ki－67 增殖指数的影响：化疗对照组、中药低剂量组、中药高剂量组与空白对照组两两比较，其 Ki－67 增殖指数均低于空白对照组（$p<0.05$）；中药低剂量组、中药高剂量组与化疗对照组两两比较，均低于化疗对照组（$p<0.05$）；而中药低剂量组及高剂量组间两两比较无明显差异（$p>0.05$）；③对 TGF－β 表达水平的影响：化疗对照组、中药低剂量组、中药高剂量组与空白对照组两两比较，空白对照组 TGF－β 表达水平明显高于前三组（$p<0.05$）；但化疗对照组、中药低剂量组与中药高剂量治疗组间两两比较，TGF－β 表达水平无明显差异（$p>0.05$）；④对 IL－10 表达水平的影响：化疗对照组、中药低剂量组、中药高剂量组与空白对照组两两比较，空白对照组 IL－10 表达水平明显高于前三组（$p<0.05$），但化疗对照组、中药低剂量治疗组及高剂量治疗组间两两比较，IL－10 表达水平无明显差异（$p>0.05$）。结论　健脾化瘀方有促进小鼠肝癌休眠的作用，其作用机制可能与其通过调节免疫抑制肿瘤细胞的 TGF－β、IL－10 表达有关。

【关键词】 健脾化瘀　肿瘤休眠　免疫调节

【Abstract】 Objective To study the effects of Jianpi Huayu formula on the promotion of tumor dormancy and the immune mechanism in mice with hepatic carcinoma by building tumor dormancy model of hepatoma in mouse intervened with Jianpi Huayu formula. **Methods** A tumor dormancy model of hepatoma in mouse was established. Mice were randomly divided into four groups: blank control group, chemotherapy control group, low－dosage Traditional Chinese medicine (TCM) group, high－dosage TCM group, intervened with normal saline, carmofur, middle－dosage Jianpi Huayu formula and high－dosage Jianpi Huayu formula respectively. After the mice were sacrificed, immunohistochemical staining was performed in the vaccinated parts extracted from mice to detect the expression of relevant indicators including ki－67, TGF－β and IL－10. **Results** ①Tumor dormancy situations: After repeated traumatic stimulation, tumor dormancy rates of blank control group and chemotherapy control group were 0 and 80.0 % respectively. The tumor dormancy rates of low－dosage and high－dosage TCM groups were both 86.7%. ②Effects on Ki－67 proliferation index: Ki－67 proliferation indexes of chemotherapy control group, low－dosage and high－dosage TCM groups were lower than that of blank control group ($p<0.05$). Ki－67 proliferation indexes of low－dosage and high－dosage TCM groups were lower than that of chemotherapy control group ($p<0.05$). There was no significant difference between Ki－67 proliferation indexes of low－dosage and high－dosage TCM groups ($p>0.05$). ③ Effects on expressions of TGF－β: The expressions of TGF－β in chemotherapy control group, low－dosage and high－dosage TCM groups were significantly lower than that in blank control group ($p<0.05$). There was no signifi－

cant difference in expressions of TGF－β between chemotherapy control group，low－dosage and high－dosage TCM groups（$p > 0.05$）. ④ Effects on the expressions of IL－10：The expressions of IL－10 in chemotherapy control group，low－dosage and high－dosage TCM groups were significantly lower than that in blank control group（$p < 0.05$）. There was no significant difference in expressions of IL－10 between chemotherapy control group，low－dosage and high－dosage TCM groups（$p > 0.05$）. **Conclusion** Jianpi Huayu formula plays a role in promoting tumor dormancy in mice with hepatic carcinoma by adjusting the immunity and suppressing the expressions of TGF－β，IL－10 in tumor cells.

【Key words】 Jianpi Huayu，Tumor dormancy，Immunoregulation

【Author´s address】 Guangdong Provincial People´s Hospital / Guangdong Academy of Medical Sciences / Guangdong Institute of Gerontology，Guangzhou 510080，Guangdong Province，China

doi:10.3969/j.issn.1671－332X.2014.09.007

肿瘤治疗的难点在于肿瘤治疗后易发生复发转移。目前研究认为，肿瘤治疗后存在休眠的肿瘤细胞，其后在一定条件下，由于机体免疫功能降低，免疫监视能力下降，肿瘤细胞通过免疫逃逸而打破休眠状态，导致肿瘤发生转移和增殖[1]。肿瘤休眠现象在临床上非常普遍，已经被许多临床及动物实验证据所证实[1－2]。逆转肿瘤细胞免疫逃逸和促进肿瘤细胞休眠方面的研究正成为近年来的研究热点[2]。

健脾化瘀法在既往研究的体内外实验中均显示出较好的抗肝癌效应。本研究拟通过建立小鼠肝癌休眠模型，并予健脾化瘀法干预，研究其促进肿瘤休眠的作用及其免疫机制，为其临床应用及进一步研究促肿瘤休眠药物提供依据。

1 材料与方法

1.1 材料

1.1.1 肿瘤细胞液 取 6～8 周龄的雄性昆明种小鼠，体重在 20～25 g，予 H22 腹水型肝癌细胞株（由中山大学动物实验中心提供）腹腔接种传代，至第 7 天，在无菌条件下，抽取腹水 3 ml，显微镜下计数细胞数为 1.0×10^8/ml。取 1 ml 腹水 +9 ml 灭菌生理盐水配制成浓度为 1.0×10^7/ml 的接种用肿瘤细胞液。

1.1.2 实验动物 选取 6～8 周龄的清洁级昆明种小鼠，体重在 20～25 g，雌雄各半，共 80 只（由中山大学实验动物中心提供，许可证号 SYXK（粤）2007－0081）。

1.1.3 药物的制备 ①中药提取液：健脾化瘀方由莪术、白术、茯苓、苦参、佛手、白花蛇舌草等组成。按组方比例取药，分别提取药物的挥发油及水溶性成分，加入适量蒸馏水，充分混匀后制成中药提取液，使每 1.0 ml 中药提取液相当于健脾化瘀方生药 1.0 g，灭菌、低温下密封保存备用，每次使用前摇匀。②卡莫氟混悬液：卡莫氟（HCFU）标准品 50 mg（由广东省药检所提供），研成均匀细末后，加入 50 ml 蒸馏水，充分混匀后配制成混悬液，浓度为 1.0 mg/ml，灭菌、低温下密封保存备用，每次使用前摇匀。

1.2 方法

1.2.1 肝癌小鼠休眠模型的建立及分组 按文中所述方法，建立肝癌小鼠休眠模型[3]。随机选出 60 只肝癌休眠模型小鼠，并按随机数字表法，将模型小鼠随机分为 4 组：空白对照组、化疗对照组、中药低剂量组、中药高剂量组，每组各 15 只模型小鼠。

1.2.2 给药方法 空白对照组：分别取 1.0 ml 生理盐水予每只小鼠灌胃，每天 1 次；化疗对照组：分别取 0.6 ml 卡莫氟混悬液（用生理盐水稀释成 1.0 ml）予每只小鼠灌胃，每天 1 次；中药低剂量组：分别取 0.3 ml 中药提取液（用生理盐水稀释成 1.0 ml）予每只小鼠灌胃，每天 1 次；中药高剂量组：分别取 0.5 ml 中药提取液（用生理盐水稀释成 1.0 ml）予每只小鼠灌胃，每天 1 次；分组后第 2 天，全部小鼠开始给药，同时，每 2 天截掉 2 mm 长的一段尾巴作为外伤刺激，重复 4 次，共给药 21 天。最后一次给药后第 2 日，以颈椎脱臼法处死全部小鼠。

1.3 标本检测

1.3.1 收集标本 处死全部小鼠后，对每只小鼠进行解剖，观察全身组织器官情况并记录，取出模型小鼠的腋下接种处组织，制备成石蜡切片以用于检测。

1.3.2 观察腋下肿瘤组织休眠情况 每日对小鼠腋下肿瘤组织情况进行观察并记录，如肉眼观察到腋下肿瘤组织生长，每日对荷瘤鼠肿瘤组织的最长径（a）及与其垂直的最短径（b）进行测量和记录，对肿瘤瘤体的体积进行计算（体积公式 $v = 1/2 \times a \times b^2$）。

1.3.3 检测 Ki－67 增殖指数（PI） Ki－67 染色阳性表达为细胞浆或核内呈棕黄色或棕褐色。在低倍镜下随机选择 5 个视野，在每个视野下换用 400 倍显微镜，计数每个视野下肿瘤细胞总数及 Ki－67 阳性增生细胞数并进行记录，每个切片至少计数 1 000 个细胞，并按以下计算公式计算出每个切片 Ki－67 增殖指数，同一组取平均值，得出每组的增殖指数。Ki－67 增殖指数按以下公式计算：Ki－67 增殖指数（PI）＝Ki－67 阳性增生细胞数/肿瘤细胞总数 ×100%。

1.3.4 TGF－β 和 IL－10 的检测 阳性表达为细胞胞质中染色呈棕黄色或棕褐色。在低倍镜下随机选择 5 个视野，在每个视野下换用 200 倍显微镜，计数每个视野下肿瘤细胞总数及阳性细胞数并进行记录，按半定量方法进行评分。表达

Results

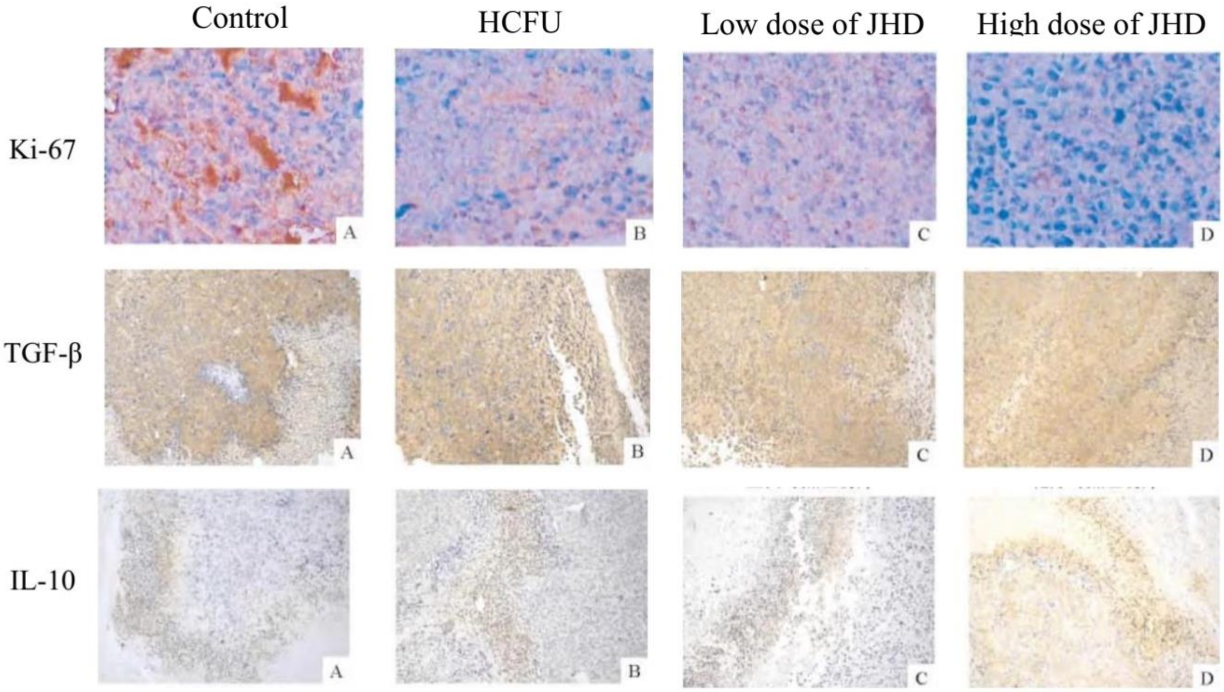

Supplementary Figure. Jianpi huayu Decoction (JHD) inhibited the expression of Ki67, TGF-β and IL-10 in tumor tissue. Representative pictures of Ki67, TGF-β and IL-10 immunohistochemical staining in tumor tissue were shown (×400 magnification). Compared with the control group, high dose of JHD significantly inhibited the expression of Ki67, TGF-β and IL- 10, and there was no significant difference with Carmofur (HCFU).
